# Dextran 500 Improves Recovery of Inflammatory Markers: An *In Vitro* Microdialysis Study

**DOI:** 10.1089/neu.2019.6513

**Published:** 2019-12-11

**Authors:** Susan Giorgi-Coll, Eric Peter Thelin, Caroline Lindblad, Tamara Tajsic, Keri L.H. Carpenter, Peter J.A. Hutchinson, Adel Helmy

**Affiliations:** ^1^ Division of Neurosurgery, Department of Clinical Neurosciences, Department of Clinical Neurosciences, University of Cambridge, Cambridge, United Kingdom.; ^2^ Department of Clinical Neuroscience, Karolinska Institutet, Stockholm, Sweden.; ^3^ Theme Neuro, Karolinska University Hospital, Stockholm, Sweden.; ^4^ Wolfson Brain Imaging Centre, Department of Clinical Neurosciences, University of Cambridge, Cambridge, United Kingdom.

**Keywords:** chemokines, cytokines, Dextran 500, *in vitro*, microdialysis, recovery

## Abstract

Cerebral microdialysis (CMD) is used in severe traumatic brain injury (TBI) in order to recover metabolites in brain extracellular fluid (ECF). To recover larger proteins and avoid fluid loss, albumin supplemented perfusion fluid (PF) has been utilized, but because of regulatory changes in the European Union, this is no longer practicable. The aim with this study was to see whether fluid, absolute (AR), and relative (RR) recovery for the novel carrier, Dextran 500, was better than conventional PF for a range of cytokines and chemokines. An *in vitro* setup mimicking conditions observed in the neurocritical care of TBI patients was used, utilizing 100-kDa molecular-weight cut-off CMD catheters inserted through a triple-lumen bolt cranial access device into an external solution with diluted cytokine standards in known concentrations for 48 h (divided into 6-h epochs). Samples were run on a 39-plex Luminex (Luminex Corporation, Austin, TX) assay to assess cytokine concentrations. We found that fluid recovery was inadequate in 50% of epochs with conventional PF, whereas Dextran PF overcame this limitation. The AR was higher in the Dextran PF samples for a majority of cytokines, and RR was significantly increased for macrophage colony-stimulating factor and transforming growth factor-alpha. In summary, Dextran PF improved fluid and cytokine recovery as compared to conventional PF and is a suitable alternative to albumin supplemented PF for protein microdialysis.

## Introduction

Cerebral microdialysis (CMD) is a technique enabling sampling from the extracellular fluid (ECF) *in vivo*, providing a unique opportunity to study underlying metabolic and inflammatory processes that occur in traumatic brain injury (TBI).^1,2^ Microdialysis sampling is based on the free diffusion of analytes across a semipermeable membrane with a nominal molecular-weight cutoff (MWCO). The membrane is attached to inlet and outlet tubing through which perfusion fluid (PF) is slowly pumped and collected.^3^ To measure metabolites in clinical practice, such as glucose, lactate, and pyruvate, a 20-kDa MWCO is adequate and an isotonic solution, mimicking cerebrospinal fluid, is used as a carrier.^4^ Microdialysis of proteins is limited by both lower absolute concentrations within the brain ECF, as well as the larger molecular weight, necessitating the use of larger MWCO membranes. This causes a number of problems, including non-specific adsorption to the device materials, clogging of membranes, and protein-protein interactions, which all negatively affect recovery.^5–7^ A further issue with increased MWCO catheters (e.g., 100 kDa) is loss by convection of fluid within the catheter.^8^ This is attributed to the hydrostatic pressure differences (with a relatively low osmotic pressure in the PF) and referred to as ultrafiltration. This may impact on both the ability to carry out analysis on the diminished volume of fluid recovered by the catheter as well as potentially impacting on the biology of the extracellular space, such that it is not representative of the underlying processes of interest. In order to mitigate this phenomenon, addition of colloid to the PF to increase the oncotic pressure has been recommended, typically albumin.^6,9^ However, a regulatory reclassification of albumin within the European Union as a blood product has made formulation of albumin-supplemented fluid logistically and financially impractical. Further, the theoretical risk of albumin leak and accumulation in the surrounding tissues has been raised, with potential negative consequences.^8^ Our group and others have shown that cytokines and chemokines are key mediators in the inflammatory processes after TBI,^10–12^ and in order to advance the study of brain protein recovery and potential therapeutic advances, an accurate estimation of relative recovery is necessary. Thus, it is imperative for the continued clinical use of microdialysis that alternative strategies for improving cytokine recovery which do not require blood products are developed and tested.

An alternative colloid to increase the osmotic and hydrostatic pressure of microdialysis PF is Dextran.^13–15^ Dextrans are branched glycans of varying molecular sizes (3–2000 kDa), of which ranges between 60 and 500 kDa have been extensively studied in the microdialysis setting.^7,9,16,17^ In comparison to normal PF, and even albumin PF, studies have shown an improved recovery of macromolecules using different molecular weights and concentrations of Dextrans.^7,9,16^ Recent *in vitro* studies have suggested that a 3% Dextran 500-kDa solution is the most suitable additive given that it is large enough not to pass through the microdialysis membrane,^18^ maintains the greatest fluid recovery,^7,14^ and does not lead to an inflammatory response in the surrounding tissue.^8^

Thus, the aim of this study was to determine whether PF supplemented with the recently commercially available 3% Dextran 500 kDa (Perfusion Fluid central nervous system [CNS] Dextran; M Dialysis, Stockholm, Sweden) could improve the fluid, absolute (AR), and relative recovery (RR) of inflammatory markers (39 cytokines and chemokines) during microdialysis sampling *in vitro*, in comparison to normal PF available for clinical use (Perfusion Fluid CNS; M Dialysis). The two types of PF were tested using an *in vitro* setup that closely approximates the clinical environment, to ascertain whether Dextran would be worthwhile to use during microdialysis sampling in human patients.

## Methods

### Materials

All high-purity deionized water (dH_2_O) used was of high-performance liquid chromatography grade (18.2 MΩ.cm, Millipore Direct Q5 UV water purification system with LC-Pak polisher; Millipore, Burlington, MA). All reagents were also of analytical grade, purchased from Sigma-Aldrich (Poole, UK), and used as received, unless otherwise stated. Sodium chloride and potassium chloride were purchased from BDH Laboratory Supplies (Poole, UK).

M Dialysis 71 CMD catheters (100-kDa nominal MWCO, polyarylethersulfone 10-mm membrane length), microdialysis vials, Perfusion Fluid CNS, Perfusion Fluid CNS Dextran, M Dialysis 106 microdialysis pumps, and corresponding batteries and syringes were purchased from M Dialysis (Stockholm, Sweden). Both PFs contain 147 mM of NaCl, 2.7 mM of KCl, 1.2 mM of CaCl_2_, and 0.85 mM of MgCl_2_, but with an additional 3% 500-kDa molecular-weight Dextran in the Perfusion Fluid CNS Dextran. This newly commercially available product was purchased from M Dialysis.

*In vitro* microdialysis sampling experiments were performed using a VWR (Radnor, PA) advanced hotplate magnetic stirrer with temperature probe. Catheters were held in place during *in vitro* sampling using a triple-lumen cranial access device (Technicam, Newton Abbott, UK). Custom Invitrogen eBioscience ProcartaPlex^TM^ human cytokine and chemokine 39-plex bead assays and human cytokine and chemokine standards (referred to by the manufacturer as “A,” “B,” “C,” “D,” “E,” “G,” “K,” “L,” and “MMP” standard mixes, plus individual standards for Galectin-3, metalloproteinase domain-containing protein [MDC], and transforming growth factor [TGF]-alpha) were purchased from Thermo Fisher (Paisley, UK). A complete list of the cytokines and chemokines analyzed is provided in Supplementary Table S1. ProcartaPlex multiplex assays were analyzed using a Luminex 200 analyzer (Luminex Corporation, Austin, TX) operating with Luminex xPONENT^®^ software. Wash steps were performed using a ProcartaPlex hand-held magnetic plate holder.

### *In vitro* microdialysis sampling

*In vitro* microdialysis sampling was performed using an artificial external solution (ES) representative of the brain extracellular environment. The ES comprised PF with 0.05% (w/v) sodium azide, 1 mg/mL of human serum albumin (HSA), and 39 human cytokines and chemokines, prepared in a 50-mL centrifuge tube (Falcon^®^) as follows. Microdialysis PF for the external solution was made in-house (147 mM of NaCl, 2.7 mM of KCl, 1.2 mM of CaCl_2_, and 0.85 mM of MgCl_2_; pH ∼6.0), to the same specifications as Perfusion Fluid CNS used for CMD in patients. The mixed cytokine and chemokine standards (A, B, C, D, E, G, K, L, and MMP standard mixes, plus individual standards for Galectin-3, MDC, and TGF-alpha), received as lyophilized powders, were resuspended in accord with the manufacturers' instructions and subsequently diluted to 1:100 in PF with 0.05% (w/v) sodium azide and 1 mg/mL of HSA (final concentration). The total volume of the ES was 25 mL. The final cytokine and chemokine concentrations are shown in Supplementary Table S1.

The centrifuge tube (50 mL) containing the ES (25 mL) was suspended using a clamp stand in a thermostatically controlled glycerol bath (to avoid condensation) set to 37°C. Very gentle agitation of the external solution was applied using a magnetic stirrer. Two M Dialysis 71 brain microdialysis catheters were placed into the external solution through a triple bolt cranial access device, which was secured within the centrifuge tube using self-adhesive plastic film. Each catheter was perfused at 0.3 μL/min using M Dialysis 107 pumps, with syringes loaded with approximately 1.5 mL of either normal PF or PF containing 3% Dextran 500. Both the normal PF and Dextran PF were used as received from the manufacturer. The microdialysate samples were collected in microdialysis vials at the end of each catheter. Pumps and collection vials were kept at the same height either side of the glycerol bath, to nullify any hydrostatic pressure differences.

**FIG. 1.**
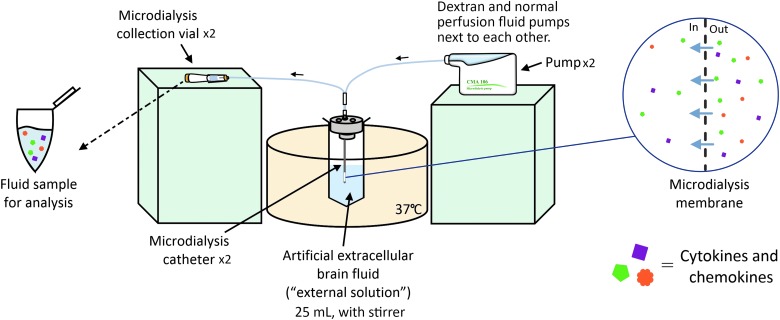
Schematic overview of the experimental setup. Color image is available online.

Sampling was performed for 48 h in total; the microdialysis vials were changed every 6 h, and sample from the ES was drawn at the 0-, 24-, and 48-h time points. A schematic of the *in vitro* sampling setup is shown in Figure 1. If no fluid was apparent in the microdialysis vial during the first 30–60 min after an exchange, that pump was flushed. This flush sequence was discarded and fluid collection started after the flush was completed. The pumps were randomly changed between fluid carriers and experiments as to not introduce any systematic bias. The *in vitro* sampling test was repeated in three independent experiments over the course of 4 weeks (eight time epochs per experiments, a total of 24 epochs). All samples were stored at −80°C before analysis.

### Sample analysis

Quantitative analysis of cytokine and chemokines was performed using custom ProcartaPlex^TM^ human 39-plex bead-based immunoassay kits. Samples were thawed and gently mixed before analysing. In total, 25 μL of sample was used per well; all samples were analyzed in duplicate. The assay was performed as per the manufacturers' instructions. Wash steps were carried out using a hand-held magnetic plate holder (ProcartaPlex). All assays were analyzed on a Luminex 200 platform. We established that Dextran 500 did not interfere with the analysis by running a standard curve using the Dextran PF as a diluent and compared it to a normal PF standard curve, and Dextran PF had no discernible effect on the standard response.

### Statistical analysis

All statistical analyses were conducted using the statistical software R, and its graphical interface Rstudio^®^ (R Foundation for Statistical Computing, Vienna, Austria).^19^ For each described analysis below, complete case analyses were conducted. A *p* value ≤0.05 was considered significant in all analyses. Graphical presentation was conducted using the R packages tidyverse,^20^ cowplot,^21^ and RColorBrewer,^22^ unless otherwise stated.

To assess how flushing the pumps affected cytokine recovery in the remaining fluid collected in that epoch, we used the R package nlme^23^ and conducted a linear mixed model per cytokine, using cytokine recovery as a dependent variable and time together with flush as independent variables.^24^ In each model, the independent experiment was considered to be the random intercept. Some cytokines (Fractalkine, interferon [IFN]-α, IFN-γ, and tumor necrosis factor [TNF] receptor type I [TNF-RI]) could not be quantified in the microdialysates, given that the concentrations recovered were below the lower limit of detection (as specified by the kit manufacturer) for the assay. Assumptions were examined graphically with regard to equal variance, linearity, and normal distribution.

Similarly, for absolute recovery analysis, time and carrier were used as independent variables in a mixed model.^23–26^ The dependent variable was the recovered cytokine value. For random intercepts, we used independent experiments. IFN-γ was excluded from analysis because the returned levels were below lowest levels of detection for both PFs. Assumptions were examined graphically as described above.^27,28^

RR was calculated as the ratio between the recovered cytokine in the microdialysis vial (numerator) and the recovered cytokine in the ES (denominator) obtained concomitantly at 24 and 48 h. For inferential analysis, cytokines exhibiting less than three positive observations (chemokine [C-C motif] ligand 20 [CCL20]/macrophage inflammatory protein 3 alpha [MIP-3α], Fractalkine, Galectin-3, IFN-α, IFN-γ, matrix metalloproteinase [MMP]-2, TNF-RI, and vascular endothelial growth factor [VEGF]-D) were excluded. For the remainders, the cytokine retrieval capacity of the different carriers (Dextran and the conventional CNS PF) were compared using a two-sided Student's *t*-test (not assuming equal variances) or (if not normally distributed) a two-sided Mann-Whitney U test.

## Results

### Fluid recovery

**FIG. 2.**
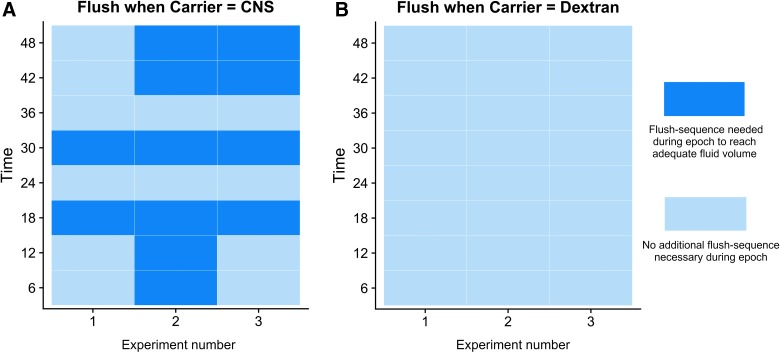
A time chart of epochs needing MD pump flushing. (**A**) Catheter perfused with Dextran 500 and (**B**) conventional (CNS) perfusion fluid. Light blue indicates an adequate fluid recovery whereas dark blue highlights an epoch where a flushing sequence was necessary given that we expected that an inadequate amount would be collected at the end of the epoch. CNS, central nervous system; MD, microdialysis. Color image is available online.

Only vials perfused with the standard PF demonstrated inadequate fluid recovery in certain epochs over the three experiments and hence needed intermittent flushing in the initial phase of the epoch (Fig. 2). In total, 50% of the vials (12 epochs) needed flushing (Fig. 2A). In these cases, sampling was briefly halted while the flush sequence was completed and resumed immediately afterward. The eluent from the flush sequence was collected separately from the sample fluid and discarded in order to avoid diluting the samples with excess fluid. For most cytokines, this procedure did not alter the absolute recovery (Table 1), but for some, notably IL-6, regulated on activation, normal T cell expressed and secreted (RANTES), and TNF, the necessary flushing sequences resulted in a significantly lower recovery in the fluid collected during the remaining of the epoch in the conventional CNS PF (Fig. 2A).

**Table 1.** Recovery of Different Cytokines

	Absolute recovery (mixed model)		Relative recovery			How flush sequence during epoch influenced AR (mixed model) Relevance of MD pump flush (mixed model)
Cytokine	If the AR significantly changed over time (*p* value)	Significant difference per carrier for AR (*p* value)	Conventional PF, mean RR in %, (SEM [%], samples)	Dextran PF, mean RR (%), (SEM [%], samples)	Significant difference (*p* value)	If significantly affected by flush sequence (*p* value)
BAFF	0.189	0.749	2.7 (0.8, *n* = 5)	2.5 (0.9, *n* = 4)	0.904	0.909
BDNF	0.175	**<0.001**	0.8 (0.3, *n* = 4)	3.8 (1.1, *n* = 5)	0.213	0.160
BLC/CXCL13	0.776	0.215	2.1 (1.2, *n* = 3)	6.8 (3.2, *n* = 2)	0.340	0.485
Eotaxin	**0.006**	**<0.001**	1.7 (0.7, *n* = 4)	8.1 (1.1, *n* = 6)	0.051	0.528
Fractalkine	**0.006**	**<0.001**	NA	NA	NA	NA
Galectin-3	0.981	0.703	NA	NA	NA	0.979
G-CSF	0.558	0.942	3.4 (1.4, *n* = 4)	2.4 (1.0, *n* = 4)	0.696	0.346
GRO-alpha	0.176	**0.004**	2.9 (1.0, *n* = 5)	2.4 (0.8, *n* = 2)	0.804	0.306
IFN-alpha	0.059	0.142	NA	NA	NA	NA
IFN-gamma	NA	NA	NA	NA	NA	NA
IL-1alpha	0.229	**<0.001**	7.7 (1.6, *n* = 6)	10.2 (1.2, *n* = 6)	0.251	0.888
IL-1beta	0.383	**0.010**	21.0 (3.4, *n* = 6)	20.7 (2.9, *n* = 6)	0.950	0.508
IL-1RA	0.148	**<0.001**	6.3 (3.2, *n* = 3)	5.8 (3.3, *n* = 2)	0.941	0.184
IL-4	0.214	**<0.001**	2.9 (0.9, *n* = 6)	7.0 (2.2, *n* = 6)	0.236	0.456
IL-6	**<0.001**	**<0.001**	0.3 (0.2, *n* = 3)	1.9 (0.4, *n* = 6)	0.076	**0.011**
IL-8	**0.005**	**<0.001**	2.5 (0.9, *n* = 5)	7.1 (1.7, *n* = 6)	0.199	0.286
IL-10	**0.024**	0.051	5.1 (3.2, *n* = 4)	3.8 (1.5, *n* = 4)	0.776	0.618
IL-12p70	**0.004**	**0.014**	0.4 (0.1, *n* = 6)	0.4 (0.1, *n* = 6)	0.782	0.361
IL-17A	**0.005**	**<0.001**	1.9 (1.5, *n* = 1)	7.2 (1.9, *n* = 5)	0.173	0.403
IL-23	0.461	0.249	2.8 (1.0, *n* = 4)	4.7 (2.2, *n* = 4)	0.589	0.233
IP-10	0.140	**<0.001**	2.4 (1.5, *n* = 4)	5.7 (1.2, *n* = 6)	0.262	0.191
MCP-1	**0.031**	**<0.001**	12.9 (2.9, *n* = 6)	25.8 (5.2, *n* = 6)	0.184	0.674
MCP-2/CCL8	**0.001**	**<0.001**	0.7 (1.2, *n* = 4)	5.5 (0.5, *n* = 6)	0.104	0.420
MCP-3	**0.004**	**0.001**	0.5 (3.7, *n* = 1)	4.7 (0.2, *n* = 2)	0.338	0.919
M-CSF	**0.004**	**0.037**	0.3 (0.1, *n* = 4)	0.8 (0.1, *n* = 5)	**0.032**	0.079
MDC/CCL22	**0.011**	**<0.001**	1.3 (0.2, *n* = 6)	4.1 (0.8, *n* = 6)	0.142	0.063
CCL3/MIP-1a	**<0.001**	**<0.001**	1.5 (1.2, *n* = 2)	10 (1.9, *n* = 6)	0.087	0.067
CCL4/MIP-1b	**0.002**	**<0.001**	2.8 (3.0, *n* = 2)	3.3 (3.3, *n* = 1)	0.912	0.078
CCL20/MIP-3a	0.322	0.503	NA	NA	NA	0.177
MMP-2	0.150	**<0.001**	NA	NA	NA	0.200
MMP-9	0.386	0.258	NA	NA	NA	0.572
RANTES	**0.003**	**<0.001**	1.4 (0.8, *n* = 3)	8.7 (1.5, *n* = 6)	0.074	**0.004**
sCD40L	0.251	0.895	13.9 (9.4, *n* = 3)	40.1 (19.4, *n* = 3)	0.439	0.244
TGF-alpha	0.970	**<0.001**	19.3 (5.2, *n* = 6)	39.6 (4.5, *n* = 6)	**0.038**	0.883
TIMP-1	**0.005**	**0.002**	0.9 (0.7, *n* = 2)	1.5 (1.8, *n* = 1)	0.728	0.158
TNF-alpha	**0.001**	**0.010**	3.3 (1.3, *n* = 4)	5.3 (2.2, *n* = 6)	0.555	**0.007**
TNF-RI	**<0.001**	**0.001**	NA	NA	NA	NA
VEGF-A	**0.009**	**0.001**	0.1 (0.1, *n* = 1)	0.6 (0.7, *n* = 2)	0.306	0.218
VEGF-D	0.195	0.854	NA	NA	NA	0.895

Table showing which cytokines that had an improved absolute recovery depending on carrier (Dextran PF or conventional PF) and which cytokines that significantly decreased over time. Mean relative recovery is shown for both the conventional PF and Dextran 500 PF, highlighting significant differences (Mann-Whitney U test). Cytokines with less than *n* = 3 detectable levels were noted as NA. Cytokines with insufficient recovered samples at 24 and 48 h could not be calculated (NA in the table). The influence that flushing the catheter system is included as well. Note that the flush eluate was not included in the actual sample for analysis. Sampling was briefly interrupted while the flush was performed (with flush eluate collected into a waste vial that was then discarded) and then sampling continued as normal afterward. Significant differences (*p* < 0.05) are in bold.

AR, absolute recovery; RR, relative recovery; MD, microdialysis; PF, perfusion fluid; SEM, standard error of the mean; NA, not available.

### Absolute recovery

**FIG. 3.**
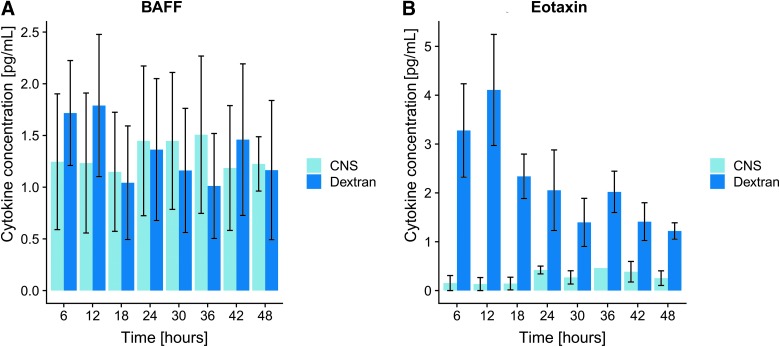
Examples of absolute recovery over time. (**A**) An example of a cytokine where the recovery did not differ significantly over time and carrier (BAFF), whereas the recovery of eotaxin (**B**) did change significantly over time or between the two carriers. The y-axis shows mean cytokine concentration (pg/mL) with standard error of mean as error bars and x-axis time (hours). BAFF, B-cell activating factor; CNS, central nervous system. Color image is available online.

Throughout the analyzed cytokines, the absolute recovery was systematically higher for the Dextran CNS PF carrier compared to the standard CNS PF (Table 1). Two examples are highlighted in Figure 3. The samples using Dextran as carrier also had more robust results overall, as visualized by Supplementary Figure S1. Further, many protein concentrations varied over time, usually with a decreasing trajectory, presumably representing a spontaneous gradual decline of some of the proteins in the study (Table 1), presumably attributed to processes such as decomposition, aggregation, and/or adhesion to surfaces, etc.

### Relative recovery

**FIG 4.**
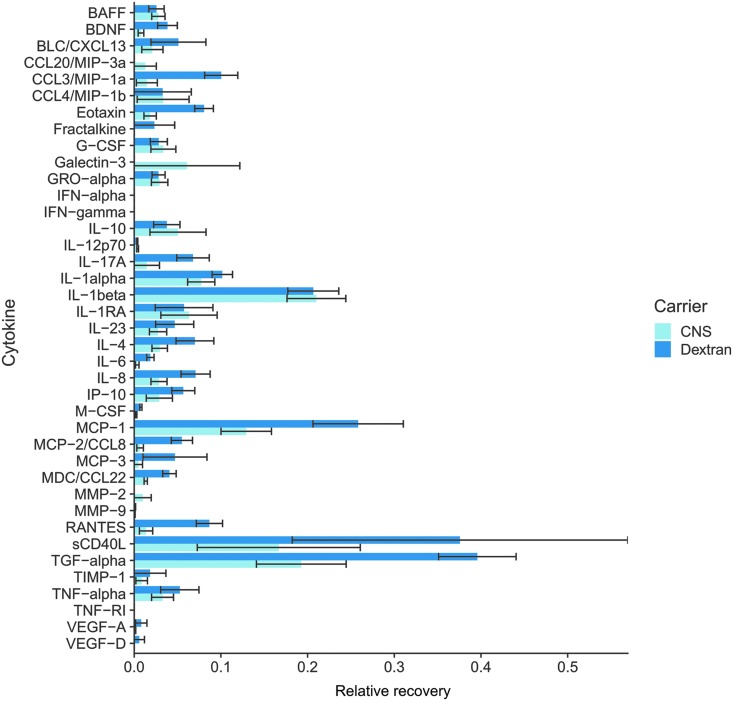
Bar plot of all relative recoveries. Mean relative recoveries (error bars represent standard error of mean) from all 3 independent experiments and pooled 24- and 48-h time points. The y-axis displays the cytokines/chemokines whereas the x-axis shows relative recovery. BAFF, B-cell activating factor; BDNF, brain-derived neurotrophic factor; BLC, B lymphocyte chemoattractant; CNS, central nervous system; CXCL13, chemokine (C-X-C motif) ligand 13; CCL, CC chemokine ligands; G-CSF, granulocyte colony-stimulating factor; GM-CSF, granulocyte-macrophage colony-stimulating factor; GRO, chemokine (C-X-C motif) ligand 1 (CXCL1); IFN, interferon; IL, interleukin; IL-1ra, interleukin-1 receptor antagonist; IP-10/IP10, interferon gamma-induced protein 10 (also known as C-X-C motif chemokine 10 [CXCL10]); MCP-1, monocyte chemotactic protein 1 (also known as CCL2); MCP-3, monocyte chemotactic protein-3 (also known as CCL7); MDC, macrophage-derived chemokine (also known as CCL22); MIP1α, macrophage inflammatory protein 1 alpha (also known as CCL3); MIP1β, macrophage inflammatory protein 1 beta (also known as CCL4); PDGF, platelet-derived growth factor; RANTES, regulated on activation, normal T cell expressed and secreted (also known as CCL5); sCD40L, soluble CD40 ligand; sIL-2Ra, soluble interleukin-2 receptor antagonist; TGF, transforming growth factor; TIMP-1, tissue inhibitor of metalloproteinase 1; TNF, tumor necrosis factor; VEGF, vascular endothelial growth factor. Color image is available online.

At 24 and 48 h, the ES was sampled to enable calculation of the RR, generating a maximum of six RRs per cytokine (two per individual experiment). Overall, there was a general trend toward a higher RR for the Dextran perfusion fluid as compared to the standard CNS (Table 1; Fig. 4). Two cytokines exhibited a significant increase in RR with Dextran PF, including M-CSF and TGF-alpha, whereas none of the cytokines' RR values were significantly higher in the standard CNS perfusion fluid.

## Discussion

To our knowledge, this is the first study using the CE-marked commercially available Perfusion Fluid CNS Dextran 500 from μDialysis, applicable for human use. This work represents a comprehensive overview of *in vitro* cytokine/chemokine recovery using this new fluid carrier as compared to the conventional CNS PF. We found that the fluid and absolute recovery were much more robust when the Dextran was used in the carrier fluid. Two cytokines also had a significantly higher RR when Dextran was used in the carrier fluid, suggesting it to be a preferable PF in comparison to conventional CNS PF.

### Dextran resulted in an improved fluid recovery

The catheters perfused with normal CNS PF needed to be “flushed” in 50% of the epochs to reach expected adequate sample volumes, as compared to the Dextran PF, which always reached sufficient volumes of recovery in the collection vials. This is presumably attributed to ultrafiltration causing a fluid loss over the membrane of CNS PF because of low osmotic pressure,^9^ though other explanations, such as varying catheter capabilities depending on the surrounding medium (*in vivo* vs. *in vitro*), have also been suggested.^6^ Similarly, in an *in vitro* recovery study by Dahlin and coworkers, they noticed a 30% fluid recovery decrease in CNS PF compared to an in-house Dextran 500 solution and only if special surface coated catheters were used (otherwise they did not observe any recovery at all),^7^ similar to Kahl and colleagues using Ringer's solution as PF.^16^ Our 50% is similar to the previous study from our group comparing 3.5% albumin with normal CNS PF, which revealed that 44% of CNS PF epochs had inadequate fluid recovery compared to none using the albumin colloid.^6^ There have been no comparisons between Dextran 500 and 3.5% albumin PF, but a study from 2005 analyzed Dextran 60 and observed that the fluid recovery was slightly better for albumin, but this difference was insignificant given that both fluid recoveries were almost 100%.^9^ It should be noted that the lower fluid volumes we noticed is not as evident for catheters with normal CNS PF placed in brain ECF *in vivo*, presumably attributed to different pressure gradients and other factors such as endogenous proteins,^6,14^ but a phenomenon more commonly observed in *in vitro* studies.^6,29^ That being said, we believe the robust fluid recovery observed for Dextran PF is translatable to the clinical scenario akin to the benefit observed with 3.5% Albumin PF.^6^ In our experience, colloid-supplemented PF mitigates against catheter failure during clinical use and reduces the need for catheter replacement. Overall, the Dextran 500 PF shows superior fluid recovery as compared to the normal CNS PF, most likely attributed to the opposing oncotic pressure generated by the colloid within the microdialysis perfusate.

### Dextran perfusion fluid improved recovery of cytokine/chemokines

We found that, overall, the Dextran PF was superior to normal CNS PF in recovery across a range of cytokines. Almost all cytokines had a significantly higher improved AR, and the RRs in the current study were significantly higher for two of the proteins, namely M-CSF and TGF-alpha, using the Dextran PF, compared to CNS PF. Although data on Dextran 500 PF from M Dialysis has never been published before in a similar fashion, Dahlin and coworkers, in Uppsala, Sweden, have escalated Dextran concentrations, and molecular weight of Dextran molecules, and noted an improved recovery of some cytokines for Dextran 500 compared to CNS PF.^7^ However, their study was not structured in a similar fashion as ours, making direct comparisons difficult; but researchers from Uppsala have now shifted to an in-house Dextran 500 as a colloid in PF and successfully recovered larger proteins in swine and rat brain injury models.^30,31^

Our group has previously performed similar *in vitro* analyses comparing a 3.5% albumin PF versus normal CNS PF,^6^ showing an RR improvement in the colloid in 9 of 12 analyzed cytokines. Further, the RRs in that study reached 30–50%, often double that of CNS PF.^6^ These RRs were higher than those observed in the current study, which revealed mean RRs of 1–10%, with some higher responders (e.g., TGF-alpha). We cannot easily explain this apparent disparity with our earlier studies, but this might be partly attributable to the different assay used and more extensive protein-protein interaction attributed to more proteins involved in the present study. In the present study, we used continuous stirring of the external solution. Comparing with the literature, some *in vitro* recovery studies utilized stirring, whereas some did not.^6,32,33^ It is conceivable that stirring may have an effect on recovery, given that the surface chemistry of interaction with a solution is a potentially complex situation in which layers and gradients can form, potentially affected by stirring. However, these lower RRs seem to be more in line with some previously published studies,^32,34–37^ where RRs in the range of 1–10% are often noted, although with some reaching higher levels. This distinct heterogeneity in results—depending on different study setups—means that caution must be exercised when comparing absolute RR between different studies.

Previous studies have shown that catheters are susceptible to biofouling over time (or even being malformed), decreasing the RR.^6,38^ We compared RRs at 24 versus 48 h, and this was not evident in our data set (data not shown), so even if protein depositions occurred they did not affect the recovery. Instead, a more probable explanation for the decrease in AR is that the ES concentrations decreased for many cytokines over time, which may be attributed to decay of the cytokines in the standard over 48 h at 37°C (Supplementary Table S1. Decreases in RR with time, and in ES concentration with time, in a different *in vitro* microdialysis setup have previously also been reported, for IL-1alpha, IL-1beta, and interleukin-1 receptor antagonist (IL-1ra).^12^

Dextran 500 and 3.5% albumin have not been compared directly in a similar fashion. However, Khan and coworkers noted that albumin is preferable to hydroxyethyl starch (HES; similar to dextran) with superior recovery for some cytokines, whereas HES was better for others, tentatively prompting investigators to a choice of PF depending on which cytokine is to be analyzed.^16^

### Future aspects and clinical implication for metabolite recovery

It is unknown what effect (if any) Dextran PF will have on the recovery of clinically analyzed metabolites (e.g., glucose, lactate, and pyruvate) as compared to normal CNS PF. A variant of Dextran 60 in PF was reportedly preferable to saline solutions in PF to recover glucose,^13^ and albumin in PF has been shown to have lower recovery of lactate compared to other colloids containing different concentrations of HES.^16^ Therefore, Dextran is presumably a preferable choice clinically as compared to albumin and normal CNS PF for the common metabolites, especially if 100-kDa catheters are used. However, before widespread clinical use, more-extensive examinations of RR of normally monitored metabolites need to be performed.

### Limitations

In the context of RR, the study was planned and designed for *n* = 6 versus *n* = 6 measurements. However, given that many cytokines were not recovered in several epochs, many of these samples returned concentrations below the lower limit of detection by the Luminex assay for this analyte. Although it could be considered a limitation to compare fewer “positive” samples, these “zero” levels presumably represent important information as to highlight which cytokines and concentrations are suitable to recover using microdialysis in the current and similar scenarios. Further, many of the recovered cytokines exhibited a higher variability than expected on the basis of our previous studies. We acknowledge that additional experimental runs would have been desirable in the present study to increase the statistical sample sizes (*n*), as previously stated. Even so, the current results shed light on the individual cytokines' different propensities for variability in recovery and thus identify those cytokine species that behave most consistently with the current microdialysis technique.

We only compared the effect of the PF in this study, whereas many other factors have been shown to alter microdialysis recovery, including membrane lengths,^13^ membrane coating,^31,39^ fluid pressure,^14^ inclusion of nanoparticles,^40^ and microdialysis pump speed,^7^ which could be other ways to improve the recovery depending on the situation. We have specifically focused on the methodological constraints within clinical practice as a prelude to utilizing this perfusion fluid in clinical studies. As mentioned above, we checked that Dextran PF did not interfere with the Luminex assay. If different analytes are to be measured by other assay techniques, it would be important to perform tests to ascertain whether or not Dextran PF interferes with those assays. For example, preliminary tests we performed with ISCUSflex measurement of glucose, lactate, and pyruvate suggested that Dextran 500 did not interfere with such measurements.

## Conclusions

*In vitro* studies are not fully representative of the situation *in vivo*, perhaps attributed to direct tissue/catheter interactions such that the outer boundary of the microdialysis catheter is in contact *in vivo* with cells, extracellular matrix, and extracellular fluid in the brain tissue, rather than a simple *in vitro* fluid solution. This makes microdialysis measures such as FR and RR a necessarily crude estimate of the recovery of a given species *in vivo*. Nevertheless, the overall benefit of Dextran PF over conventional PF for the recovery of cytokines and chemokines is supported by these *in vitro* results, and we therefore regard Dextran PF as showing promise as a perfusion fluid for use in clinical microdialysis studies requiring recovery of protein.

## Supplementary Material

Supplemental data

Supplemental data
