# Supplementary material for: Dextran 500 Improves Recovery of Inflammatory Markers: An In Vitro Microdialysis Study
Source: J Neurotrauma. 2019 Dec 11;37(1):106–14. doi: 10.1089/neu.2019.6513 (PMC6921287; doi:10.1089/neu.2019.6513)
Supplement: Supplemental data [file Supp_TableS1.pdf]

Supplementary Data

Supplementary Table S1. Concentrations of the 39 Cytokine and Chemokine Standards Sampled for During the *In Vitro* Tests

Cytokine/chemokine	*Nominal prepared concentration (pg/mL)*[a]	*Measured concentration in external solution at 0 h (pg/mL) average (SD)*	*Measured concentration in external solution at 24 h (pg/mL) average (SD)*	*Measured concentration in external solution at 48 h (pg/mL) average (SD)*
BAFF	108.0	50.177 (11.341)	47.197 (8.540)	48.398 (4.424)
BLC/CXCL13	316.0	282.571 (106.391)	274.971 (97.543)	262.187 (34.712)
BDNF	87.5	45.816 (10.955)	24.553 (7.385)	19.730 (6.123)
Eotaxin	21.5	32.225 (20.525)	24.038 (12.366)	17.737 (7.030)
Fractalkine	50.0	35.104 (11.497)	31.160 (6.610)	31.812 (1.558)
Galectin	20,831.0	6,647.821 (3713.481)	5,080.999 (3452.216)	5,038.725 (4747.579)
G-CSF	617.0	464.514 (88.902)	353.822 (50.532)	243.957 (25.829)
GRO-alpha	87.0	51.446 (25.579)	50.654 (23.137)	50.036 (19.066)
IFN-alpha	21.5	8.117 (4.824)	7.961 (3.207)	8.147 (3.420)
IFN-gamma	477.0	456.393 (103.740)	364.831 (65.945)	293.613 (78.400)
IL-1alpha	29.0	13.993 (5.343)	13.198 (5.100)	13.017 (2.866)
IL-1beta	87.5	146.881 (40.944)	131.799 (23.196)	143.907 (17.585)
IL-1ra	1398.0	800.036 (322.415)	746.143 (315.071)	741.104 (266.315)
IL-4	394.0	403.225 (165.275)	348.409 (79.662)	338.300 (57.125)
IL-6	305.0	324.075 (173.994)	356.508 (89.722)	404.397 (58.409)
IL-8	99.0	62.724 (36.237)	61.029 (30.667)	61.396 (19.943)
IL-10	81.0	95.6 (35.140)	39.416 (13.029)	18.638 (6.048)
IL-12p70	473.0	366.436 (131.735)	335.956 (56.015)	367.014 (15.020)
IL-17alpha	76.0	88.976 (26.174)	82.561 (18.122)	77.266 (10.988)
IL-23	700.0	710.517 (248.357)	389.147 (114.146)	364.311 (81.302)
IP-10	47.0	72.045 (39.951)	62.916 (33.120)	59.695 (27.177)
M-CSF	647.0	688.654 (168.870)	713.025 (142.048)	768.805 (44.494)
MCP-1	152.0	83.141 (46.870)	80.994 (40.623)	87.015 (32.790)
MCP-2	19.0	11.197 (3.467)	10.673 (2.079)	10.443 (0.179)
MCP-3	151.0	89.629 (37.017)	87.233 (29.652)	89.745 (21.667)
MDC/CCL22	595.0	300.129 (51.464)	299.409 (58.709)	308.758 (41.573)
MIP-1alpha/CCL3	72.5	44.952 (26.068)	37.378 (20.152)	34.353 (13.062)
MIP-1beta/CCL4	315.0	114.215 (64.053)	31.468 (8.633)	27.836 (4.392)
MIP-3alpha/CCL20	266.0	512.478 (197.952)	362.796 (88.812)	395.118 (84.928)
MMP-2	1,467.0	791.681 (216.760)	803.264 (168.265)	1018.612 (225.953)
MMP-9	29.5	37.720 (7.958)	37.794 (6.441)	39.537 (3.804)
RANTES	34.50	24.966 (15.245)	23.402 (10.979)	23.152 (7.614)
sCD40L	105.0	100.381 (20.923)	10.471 (3.297)	5.034 (2.877)
TGF-alpha	180.0	99.790 (28.304)	104.240 (5.983)	107.166 (9.935)
TIMP-1	1,970.0	1,010.039 (261.606)	879.430 (88.291)	1,027.716 (325.963)
TNF-alpha	396.0	482.134 (120.243)	181.313 (37.661)	86.458 (19.316)
TNF-RI	7,744.0	4,199.768 (435.351)	4,020.64 (207.650)	4,225.314 (233.140)
VEGF-A	236.0	206.361 (47.149)	208.576 (44.317)	249.192 (19.312)
VEGF-D	113.0	70.246 (19.219)	62.262 (15.665)	60.329 (9.511)

The standards were diluted to concentrations that reflect those found in the brain's extracellular fluid as shown in previous experiments from our group,[10,12] assuming a 20% relative recovery (RR).[6] Additionally, we presented the actual, measured concentrations of the external solution at time points 0, 24, and 48 h.

[a]Concentration prepared by assuming the weight of each cytokine (micrograms per ampoule) as supplied and specified by the manufacturer, and then diluted in an appropriate volume of external solution.

BAFF, B-cell activating factor; BDNF, brain-derived neurotrophic factor; BLC, B lymphocyte chemoattractant; CXCL13, chemokine (C-X-C motif) ligand 13; CCL, CC chemokine ligands; G-CSF, granulocyte colony-stimulating factor; GM-CSF, granulocyte-macrophage colony-stimulating factor; GRO, chemokine (C-X-C motif) ligand 1 (CXCL1); IFN, interferon; IL, interleukin; IL-1ra, interleukin-1 receptor antagonist; IP-10/IP10, interferon gamma-induced protein 10 (also known as C-X-C motif chemokine 10 [CXCL10]); MCP-1, monocyte chemotactic protein 1 (also known as CCL2); MCP-3, monocyte chemotactic protein-3 (also known as CCL7); MDC, macrophage-derived chemokine (also known as CCL22); MIP1α, macrophage inflammatory protein 1 alpha (also known as CCL3); MIP1β, macrophage inflammatory protein 1 beta (also known as CCL4); PDGF, platelet-derived growth factor; RANTES, regulated on activation, normal T cell expressed and secreted (also known as CCL5); sCD40L, soluble CD40 ligand; sIL-2Ra, soluble interleukin-2 receptor antagonist; TGF, transforming growth factor; TIMP-1, tissue inhibitor of metalloproteinase 1; TNF, tumor necrosis factor; VEGF, vascular endothelial growth factor.
