# Supplementary material for: Dextran 500 Improves Recovery of Inflammatory Markers: An In Vitro Microdialysis Study
Source: J Neurotrauma. 2019 Dec 11;37(1):106–14. doi: 10.1089/neu.2019.6513 (PMC6921287; doi:10.1089/neu.2019.6513)
Supplement: Supplemental data [file Supp_FigureS1.pdf]

SUPPLEMENTARY FIG. S1. Absolute recovery data for all included cytokines/ chemokines. Y-axis shows mean cytokine con-centration (pg/ml) with standard error of mean as error bars and x-axis time (hours).

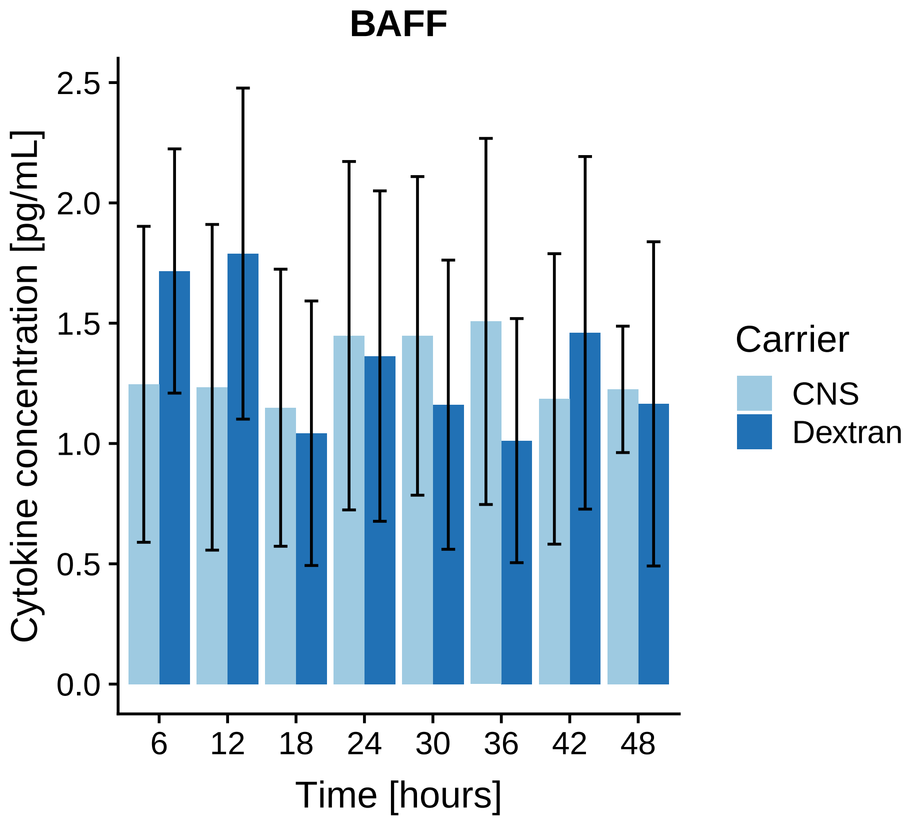
BAFF
Cytokine concentration [pg/mL]
0.0
0.5
1.0
1.5
2.0
2.5
6
12
18
24
30
36
42
48
Time [hours]
Carrier
CNS
Dextran

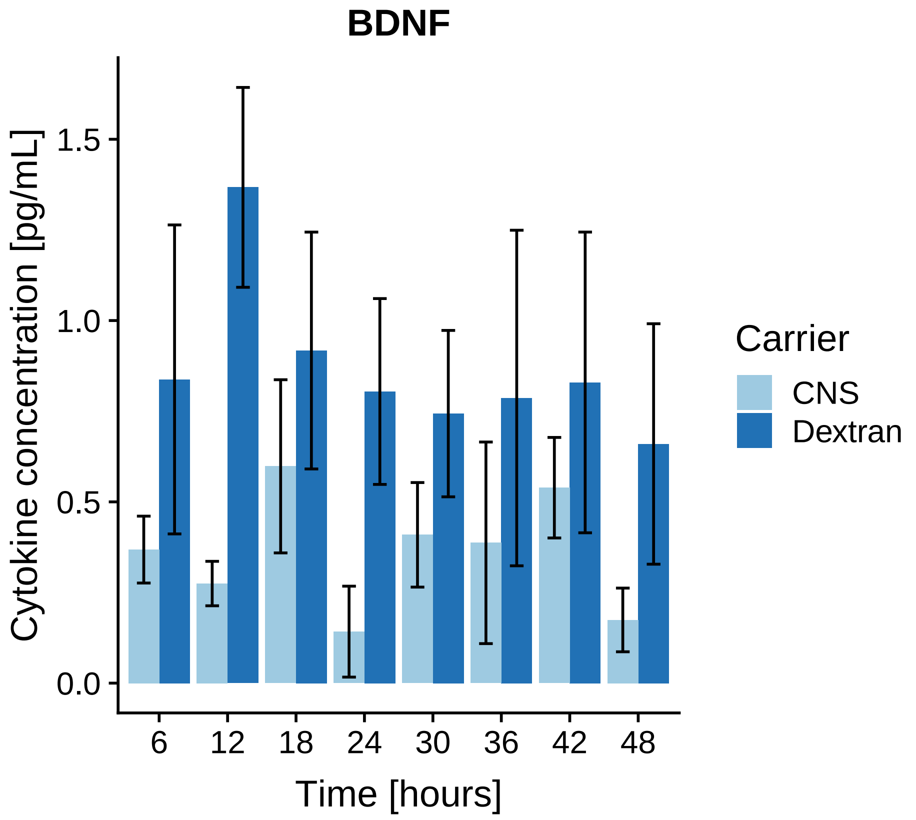
BDNF
Cytokine concentration [pg/mL]
0.0
0.5
1.0
1.5
6
12
18
24
30
36
42
48
Time [hours]
Carrier
CNS
Dextran

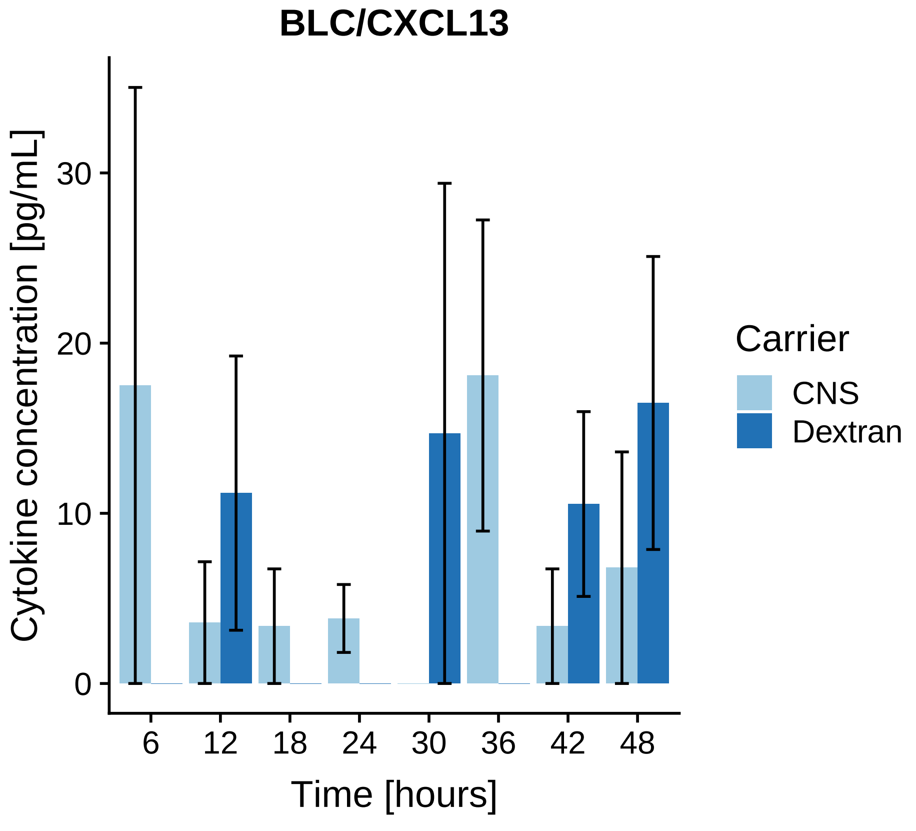
BLC/CXCL13
Cytokine concentration [pg/mL]
0
10
20
30
6
12
18
24
30
36
42
48
Time [hours]
Carrier
CNS
Dextran

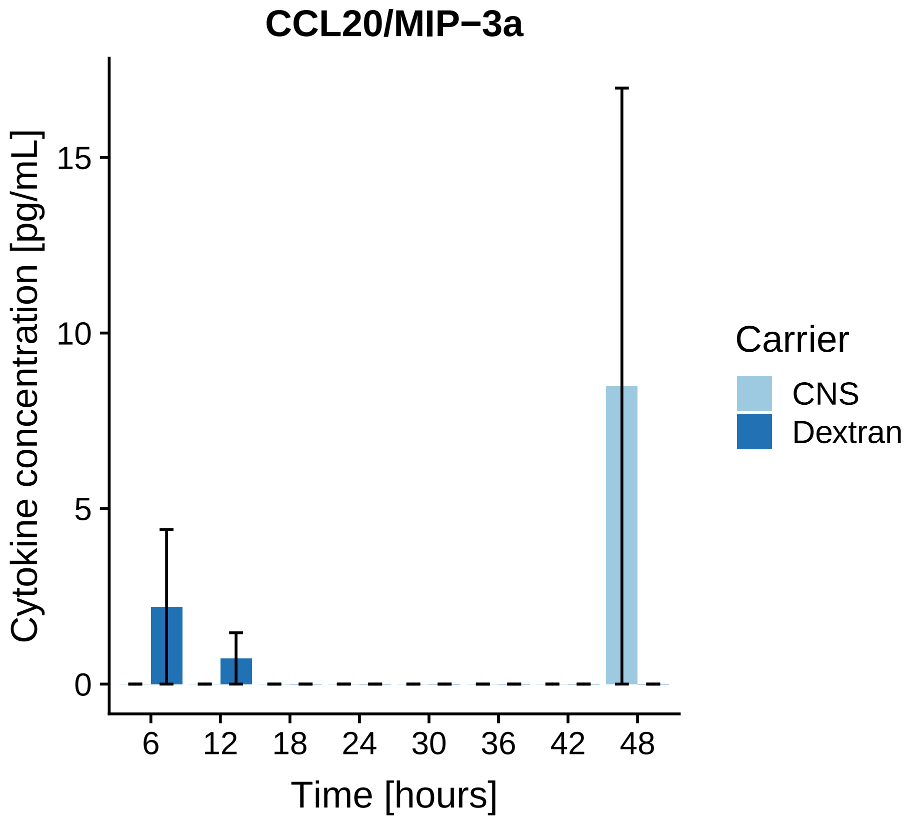
CCL20/MIP−3a
Cytokine concentration [pg/mL]
0
5
10
15
6
12
18
24
30
36
42
48
Time [hours]
Carrier
CNS
Dextran

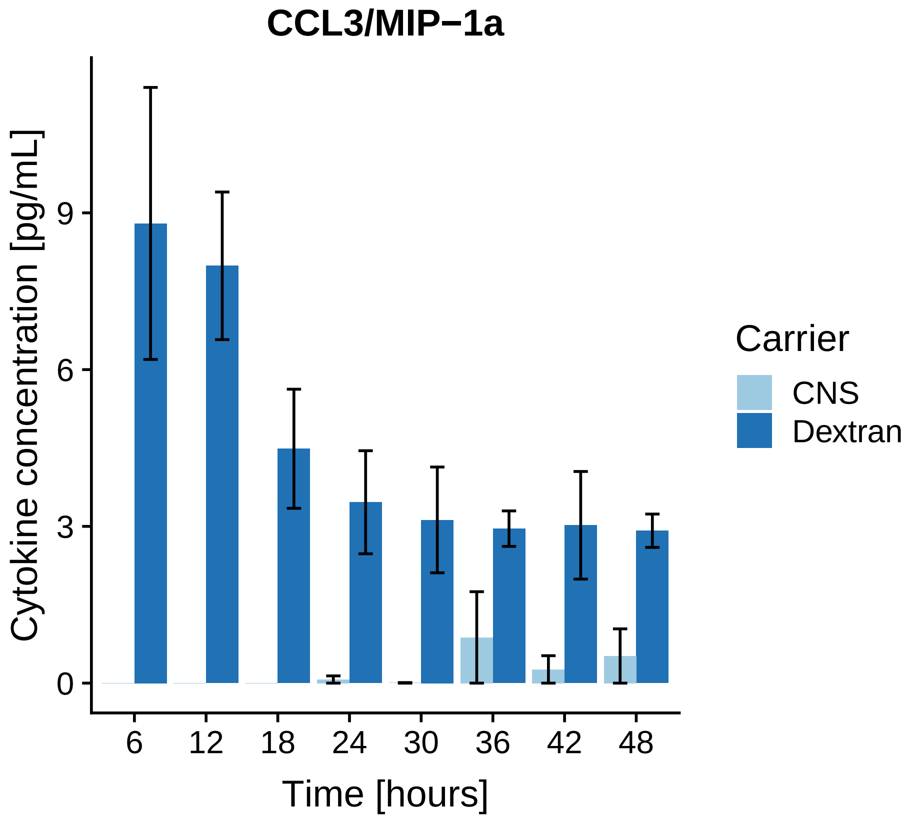
CCL3/MIP−1a
Cytokine concentration [pg/mL]
0
3
6
9
6
12
18
24
30
36
42
48
Time [hours]
Carrier
CNS
Dextran

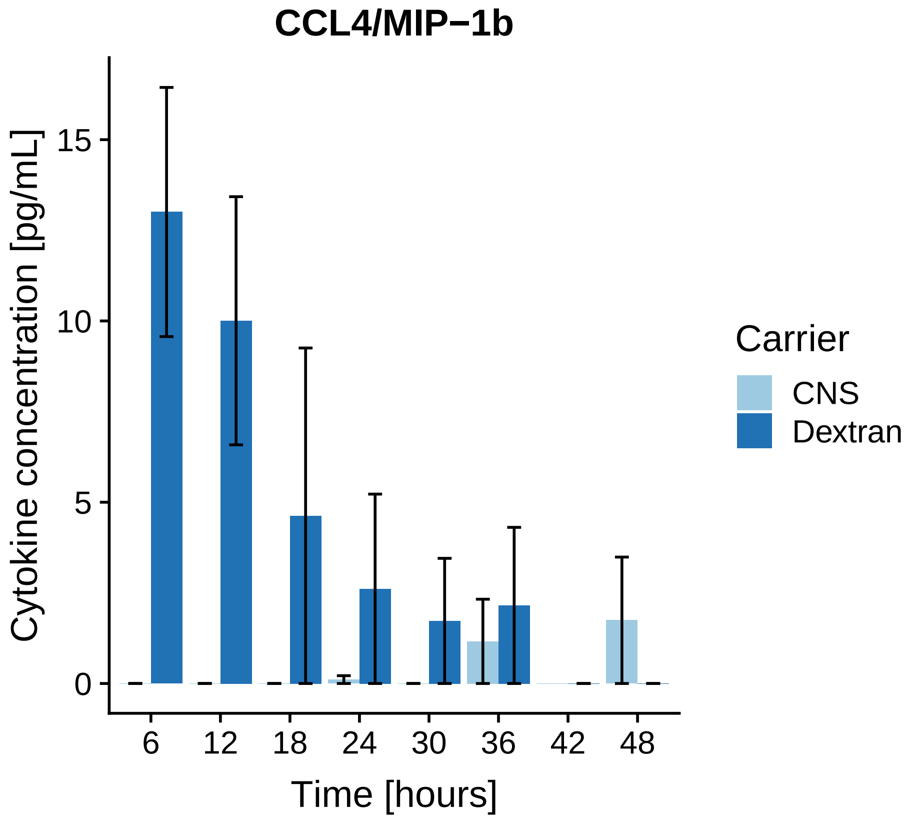
CCL4/MIP−1b
Cytokine concentration [pg/mL]
0
5
10
15
6
12
18
24
30
36
42
48
Time [hours]
Carrier
CNS
Dextran

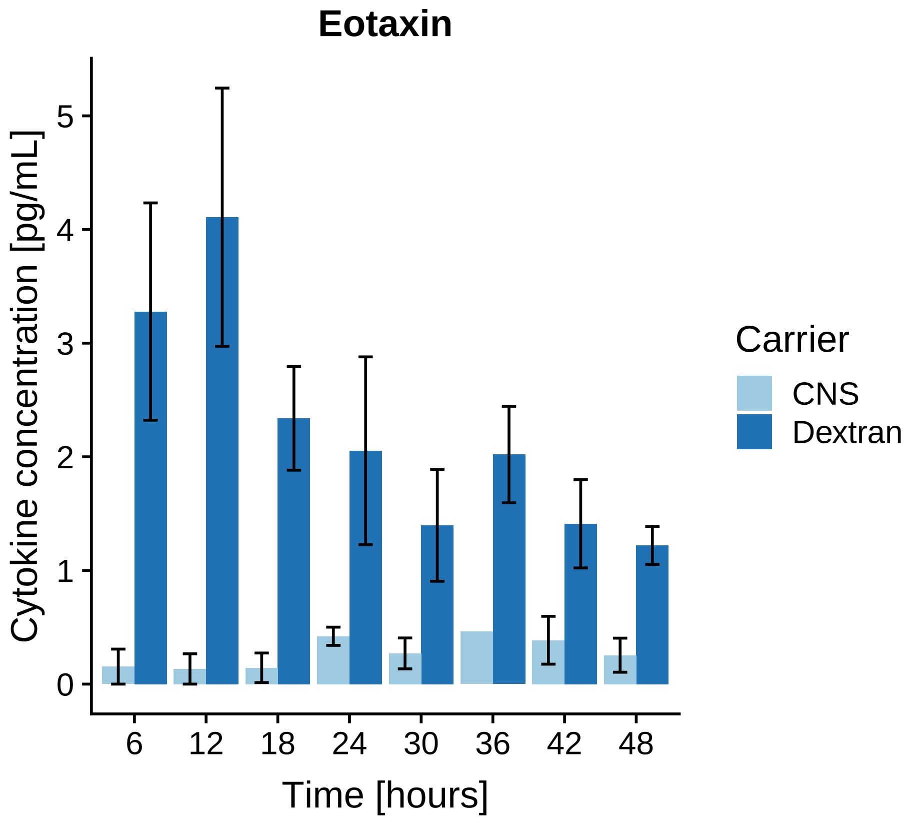
Eotaxin
Cytokine concentration [pg/mL]
0
1
2
3
4
5
6
12
18
24
30
36
42
48
Time [hours]
Carrier
CNS
Dextran

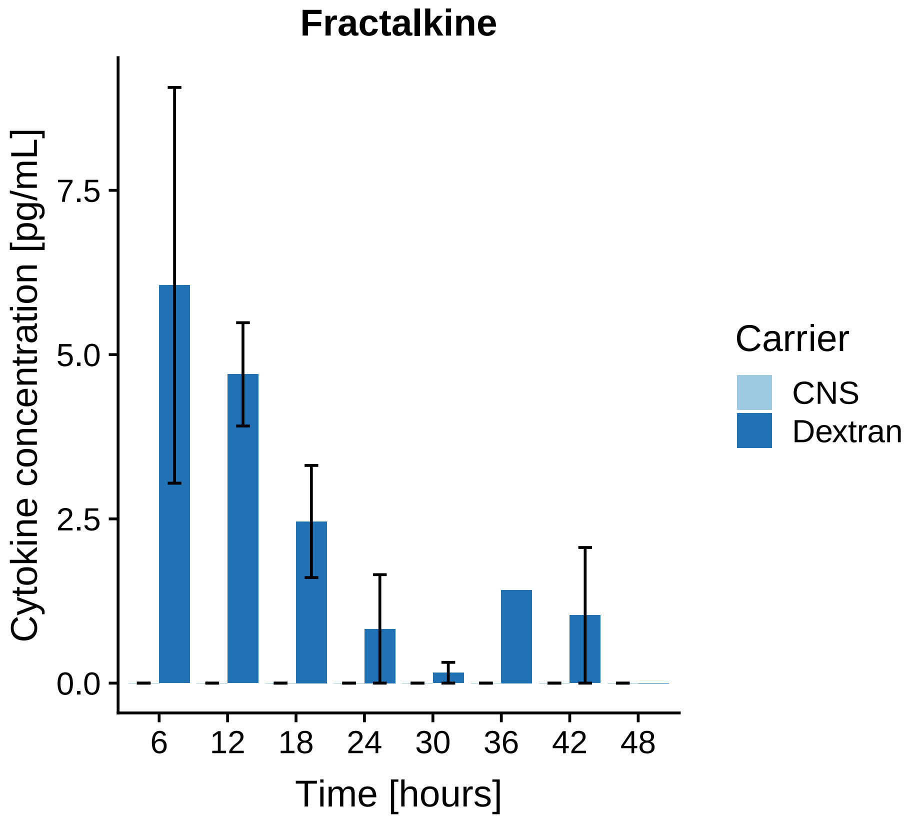
Fractalkine
Cytokine concentration [pg/mL]
0.0
2.5
5.0
7.5
6
12
18
24
30
36
42
48
Time [hours]
Carrier
CNS
Dextran

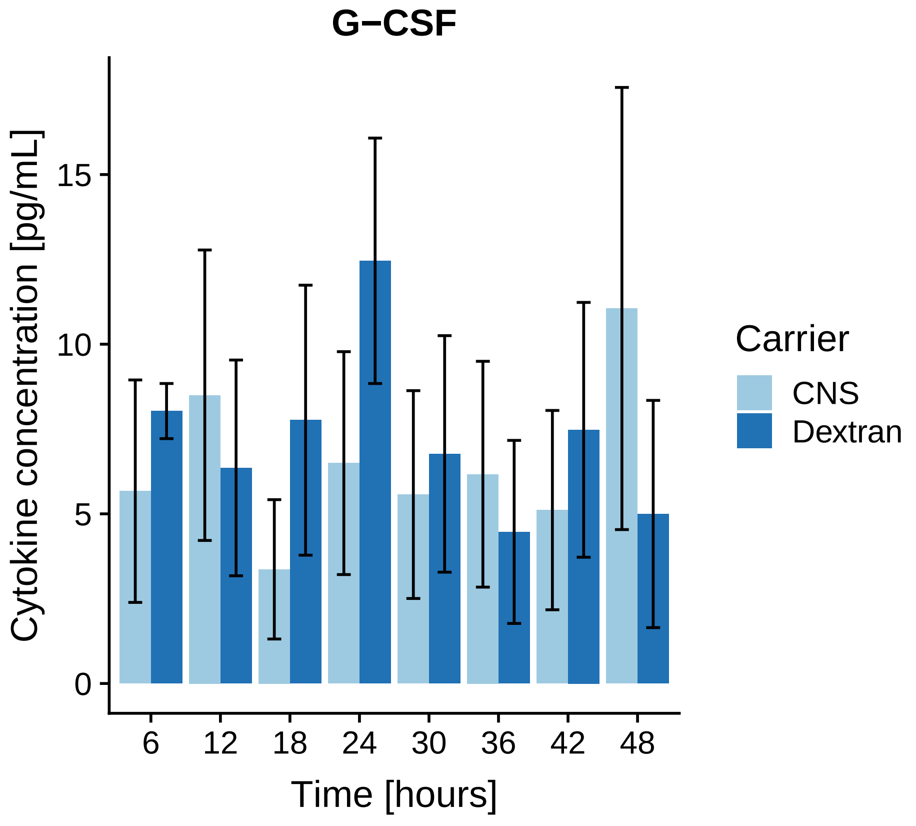
G−CSF
Cytokine concentration [pg/mL]
0
5
10
15
6
12
18
24
30
36
42
48
Time [hours]
Carrier
CNS
Dextran

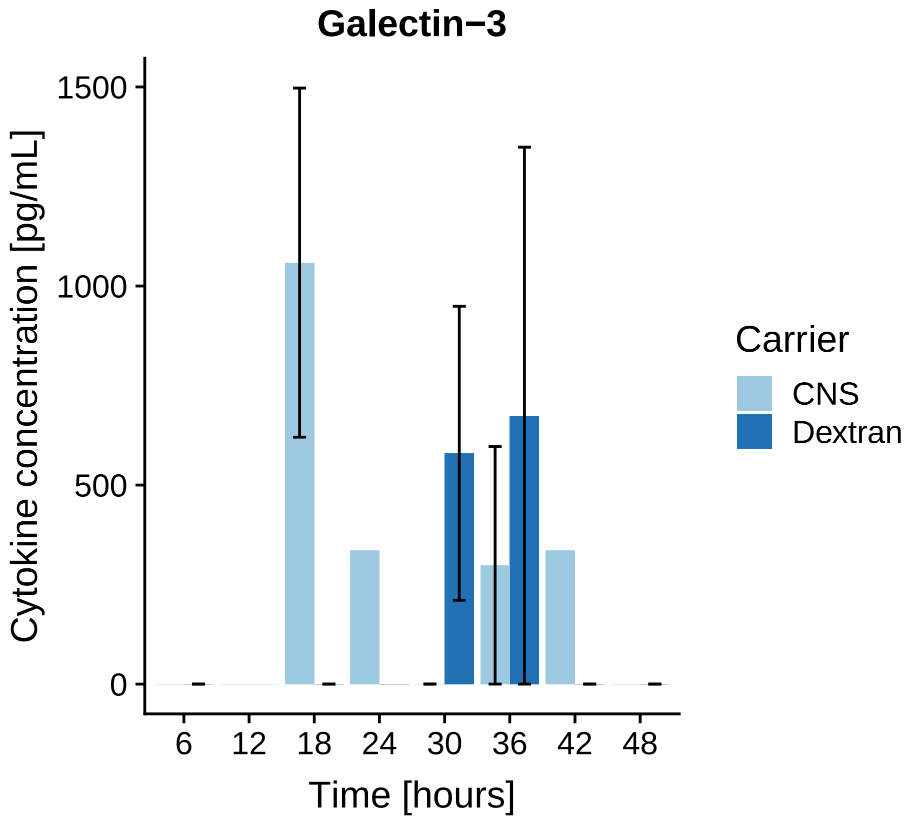
Galectin−3
Cytokine concentration [pg/mL]
1500
1000
500
0
6
12
18
24
30
36
42
48
Time [hours]
Carrier
CNS
Dextran

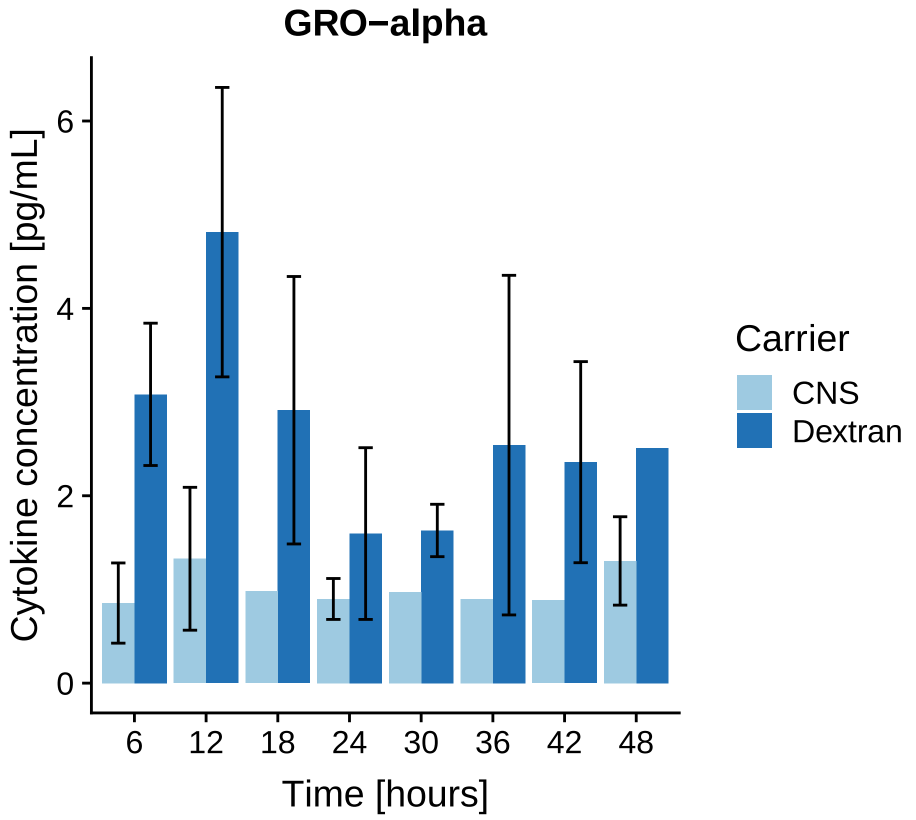
GRO−alpha
Cytokine concentration [pg/mL]
6
4
2
0
6
12
18
24
30
36
42
48
Time [hours]
Carrier
CNS
Dextran

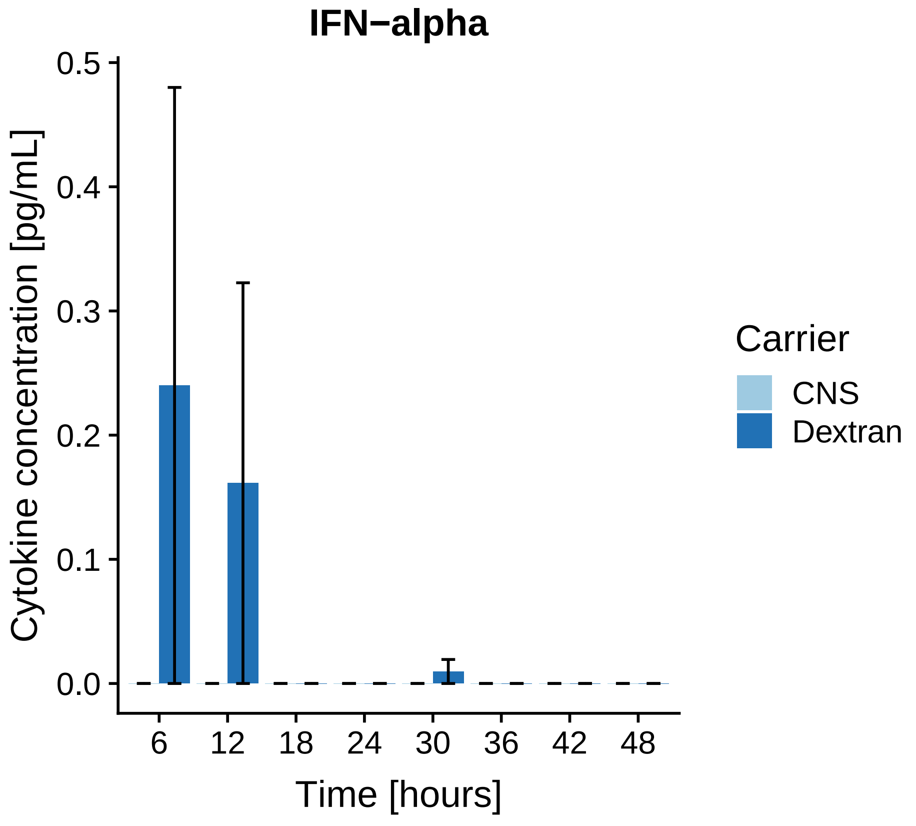
IFN−alpha
Cytokine concentration [pg/mL]
0.5
0.4
0.3
0.2
0.1
0.0
6
12
18
24
30
36
42
48
Time [hours]
Carrier
CNS
Dextran

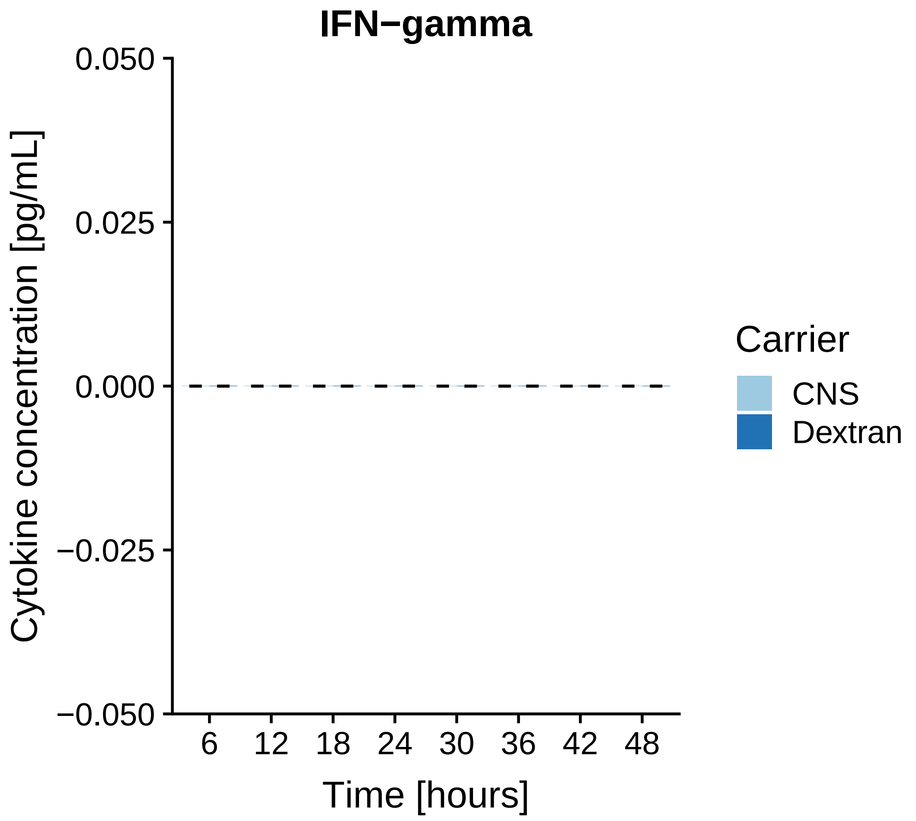
IFN−gamma
0.050
0.025
0.000
−0.025
−0.050
Cytokine concentration [pg/mL]
6
12
18
24
30
36
42
48
Time [hours]
Carrier
CNS
Dextran

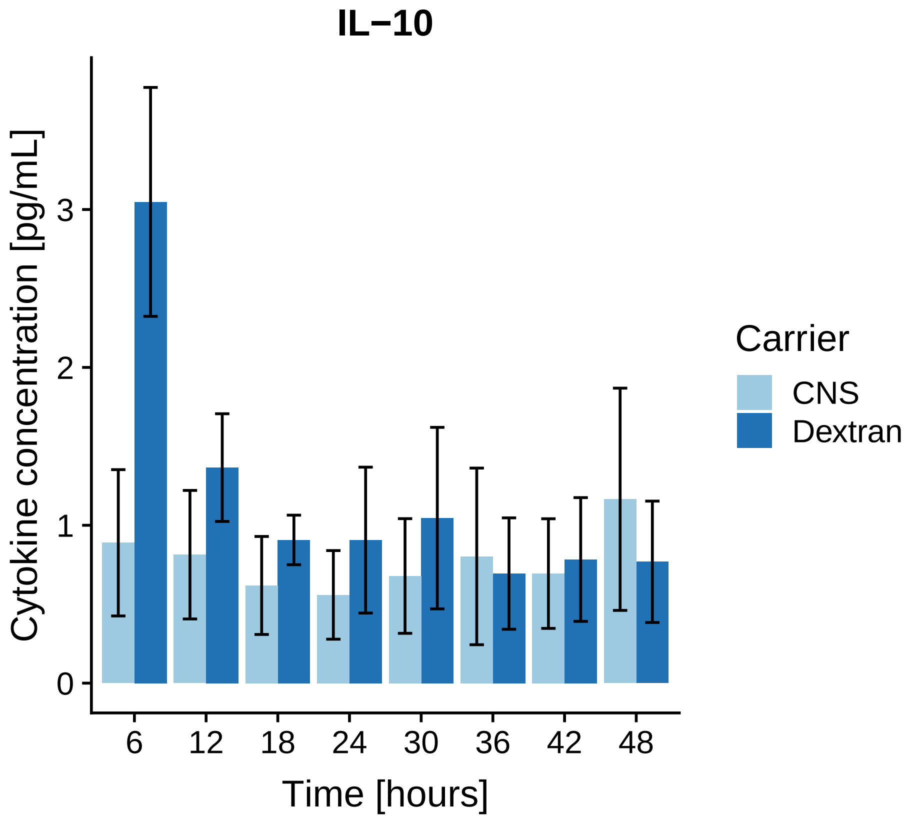
IL−10
0
1
2
3
Cytokine concentration [pg/mL]
6
12
18
24
30
36
42
48
Time [hours]
Carrier
CNS
Dextran

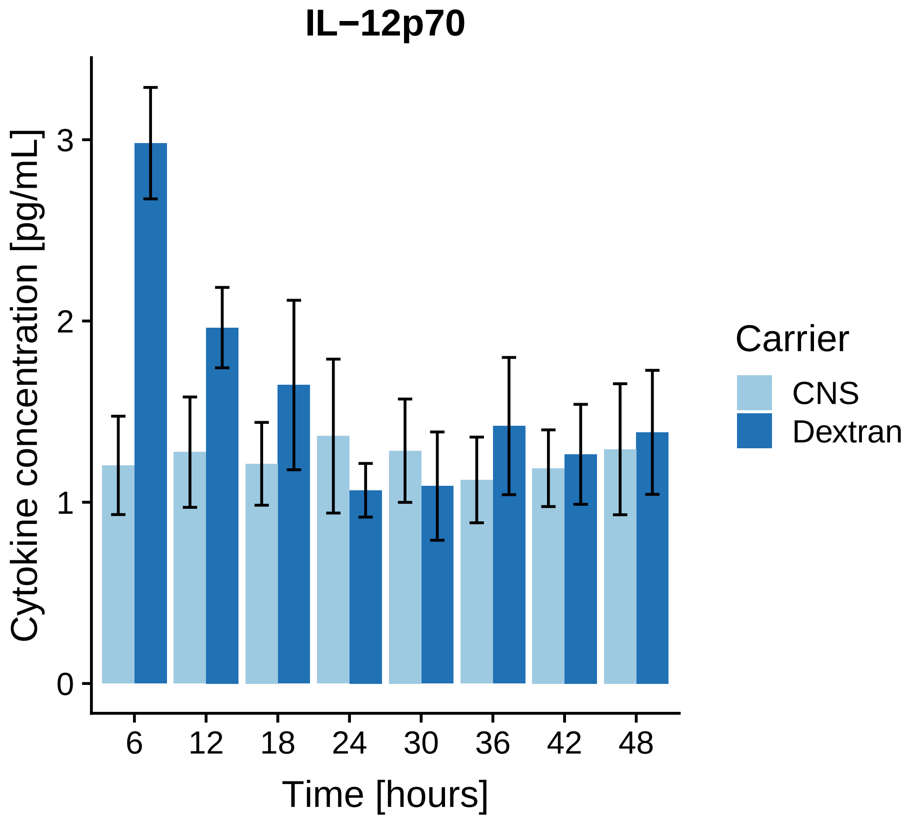
IL−12p70
0
1
2
3
Cytokine concentration [pg/mL]
6
12
18
24
30
36
42
48
Time [hours]
Carrier
CNS
Dextran

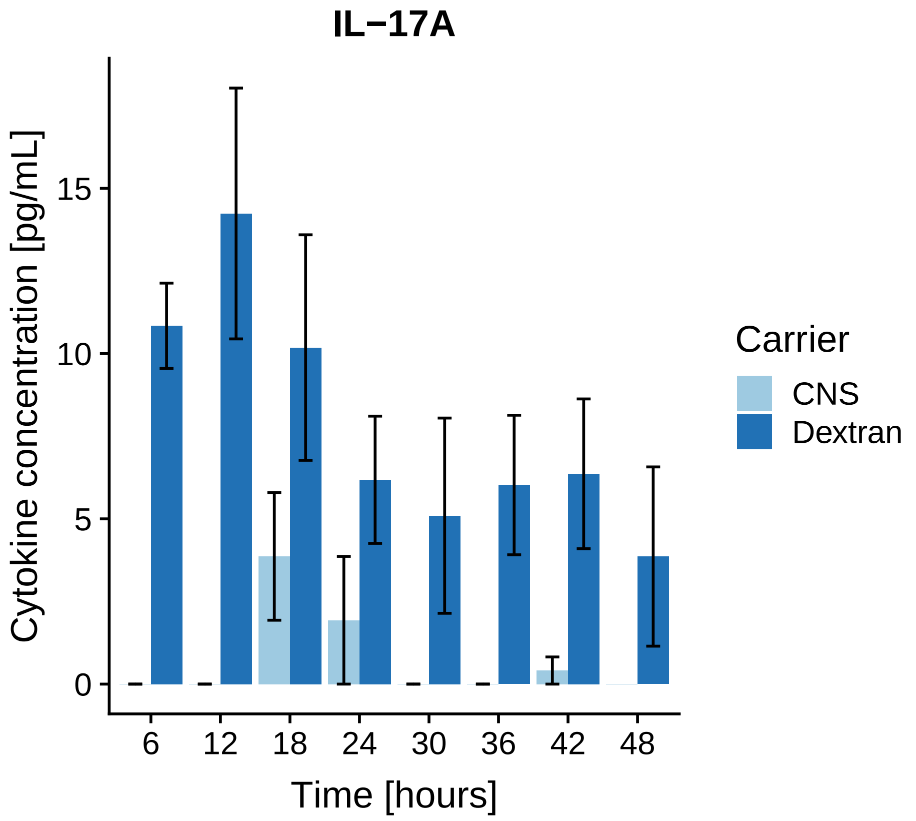
IL−17A
Cytokine concentration [pg/mL]
0
5
10
15
6
12
18
24
30
36
42
48
Time [hours]
Carrier
CNS
Dextran

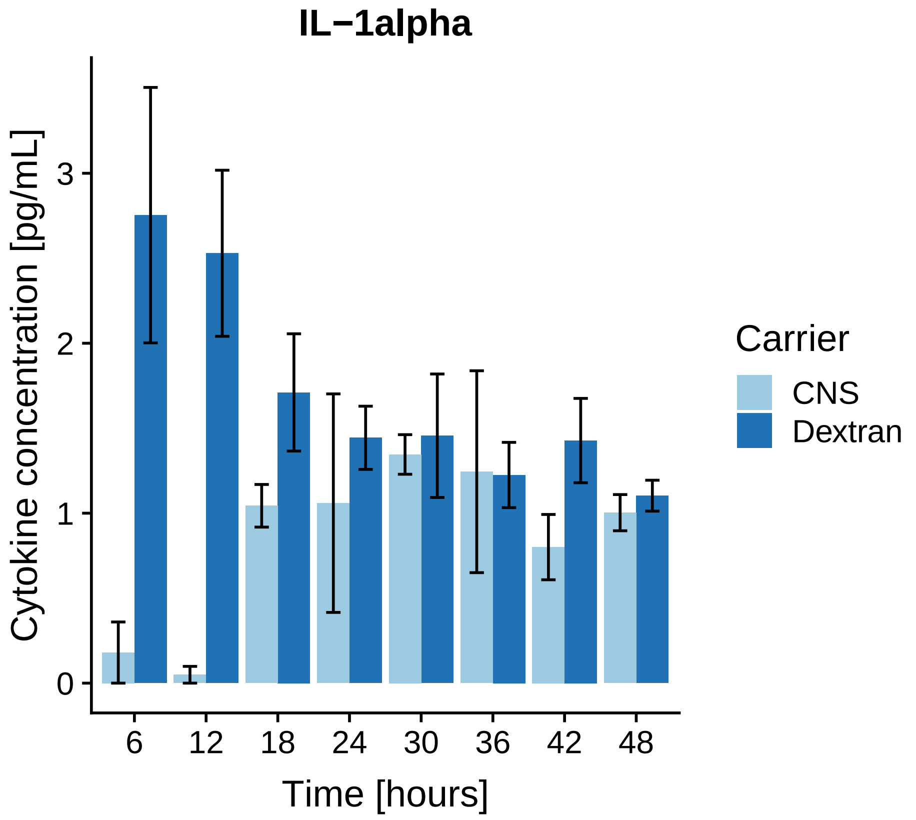
IL−1alpha
Cytokine concentration [pg/mL]
0
1
2
3
6
12
18
24
30
36
42
48
Time [hours]
Carrier
CNS
Dextran

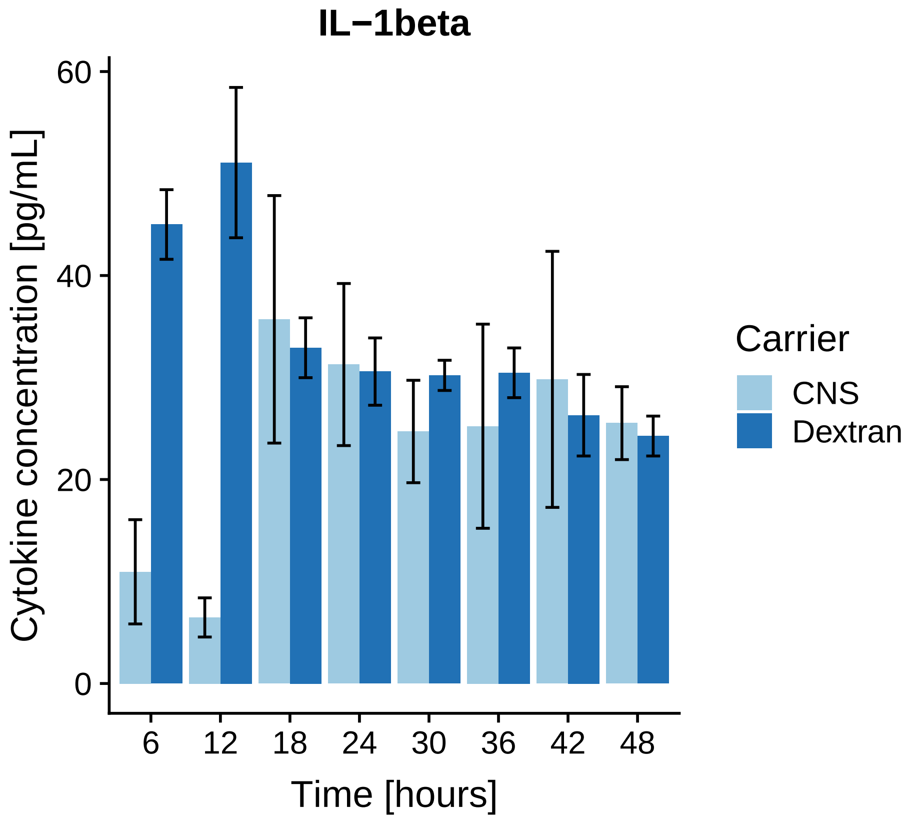
IL−1beta
Cytokine concentration [pg/mL]
0
20
40
60
6
12
18
24
30
36
42
48
Time [hours]
Carrier
CNS
Dextran

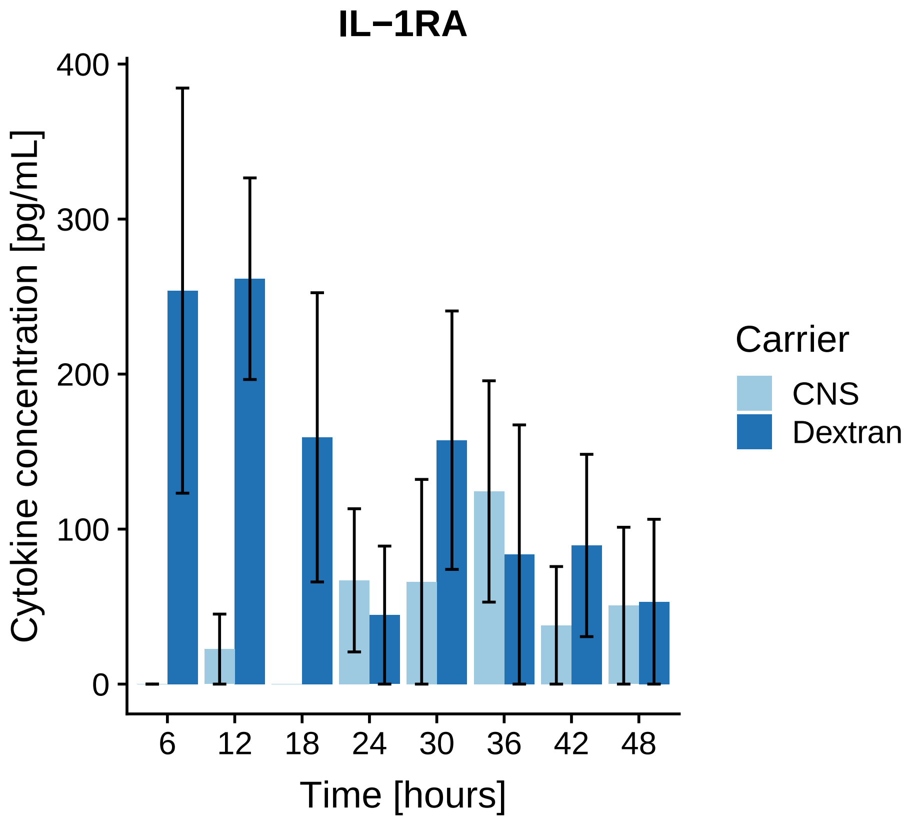
IL−1RA
Cytokine concentration [pg/mL]
400
300
200
100
0
6
12
18
24
30
36
42
48
Time [hours]
Carrier
CNS
Dextran

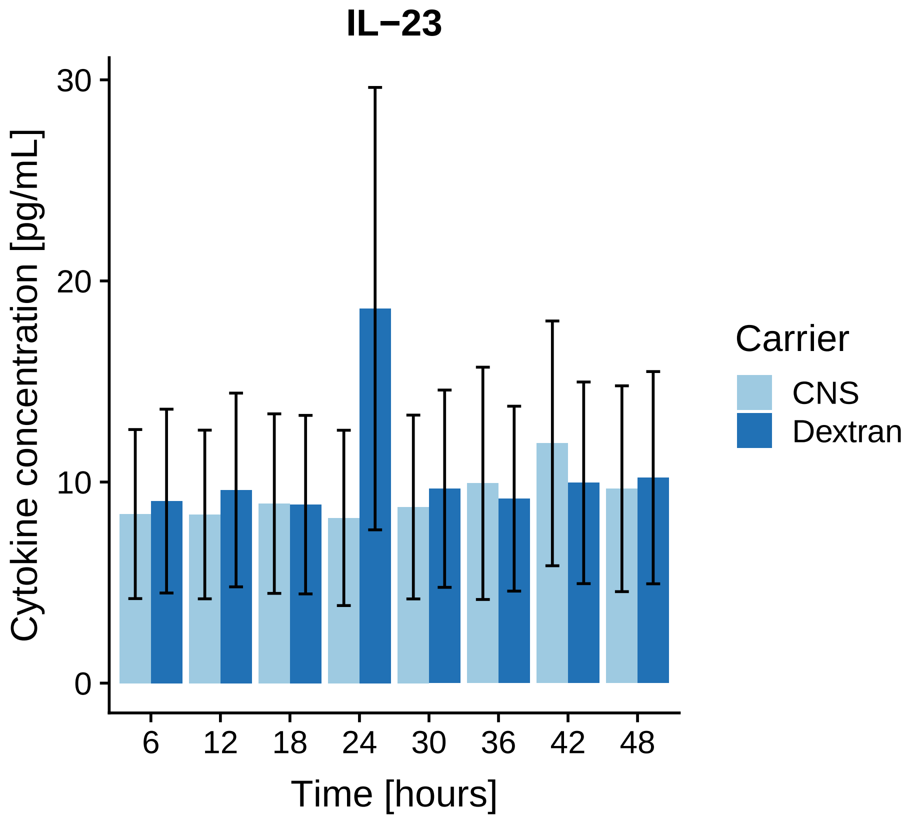
IL−23
Cytokine concentration [pg/mL]
30
20
10
0
6
12
18
24
30
36
42
48
Time [hours]
Carrier
CNS
Dextran

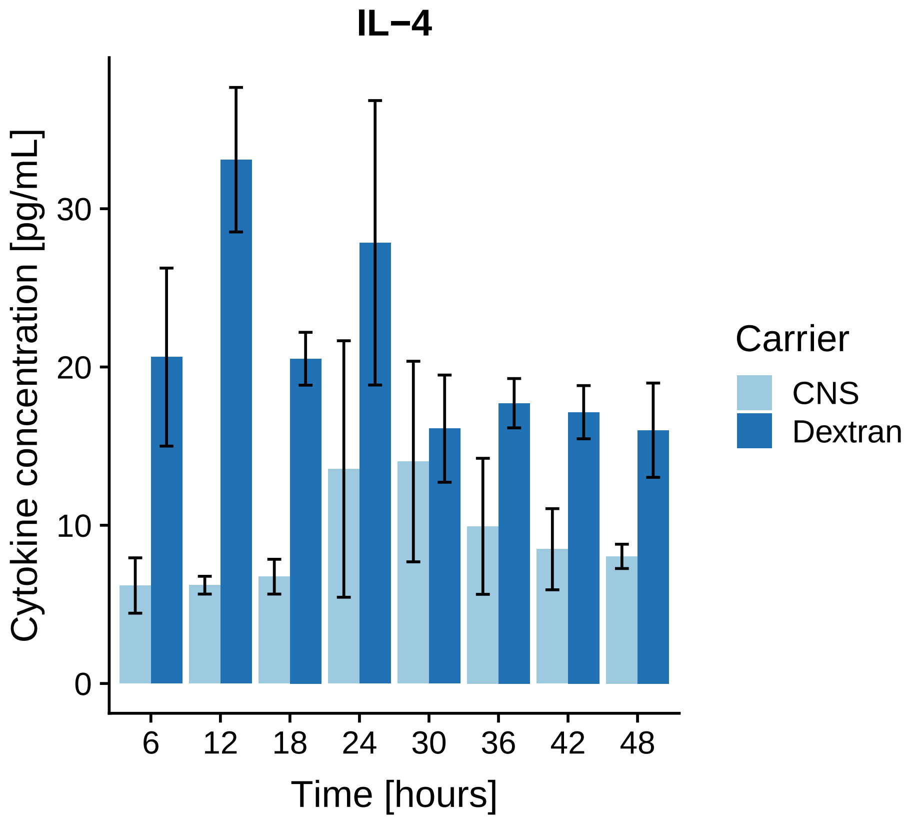
IL−4
Cytokine concentration [pg/mL]
30
20
10
0
6
12
18
24
30
36
42
48
Time [hours]
Carrier
CNS
Dextran

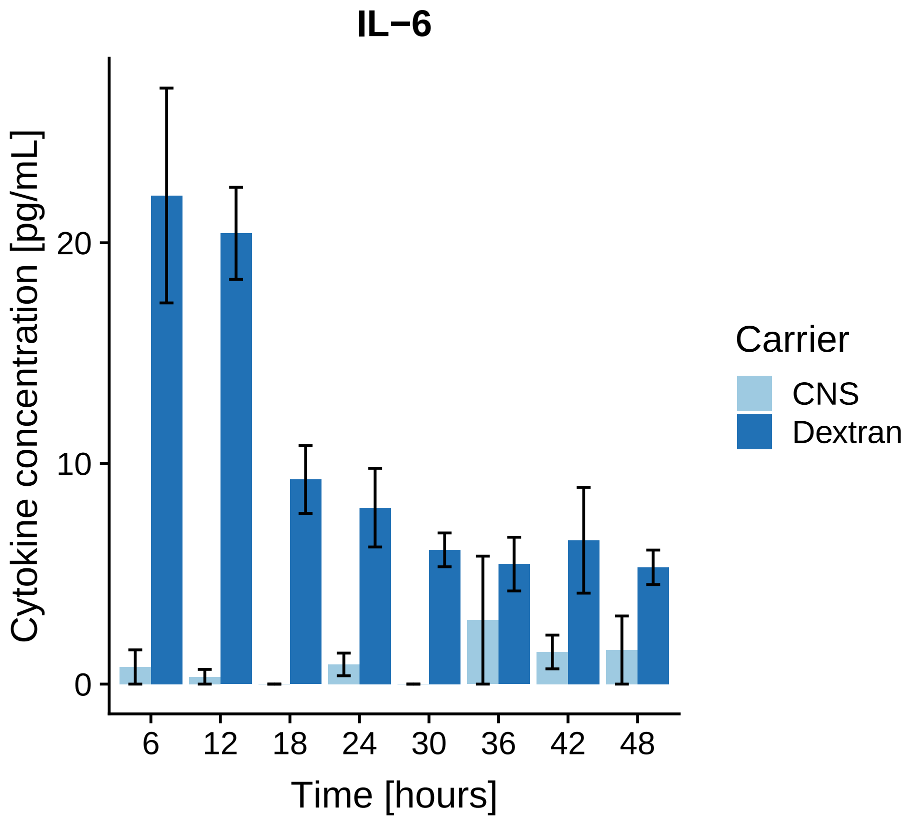
IL−6
Cytokine concentration [pg/mL]
0
10
20
6
12
18
24
30
36
42
48
Time [hours]
Carrier
CNS
Dextran

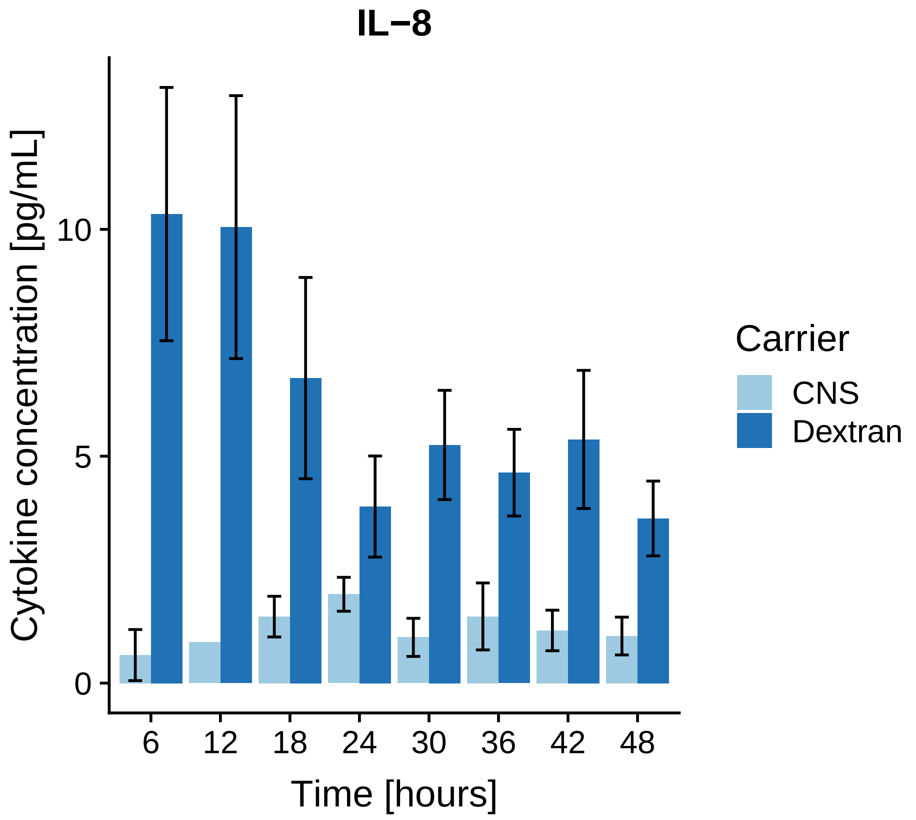
IL−8
Cytokine concentration [pg/mL]
0
5
10
6
12
18
24
30
36
42
48
Time [hours]
Carrier
CNS
Dextran

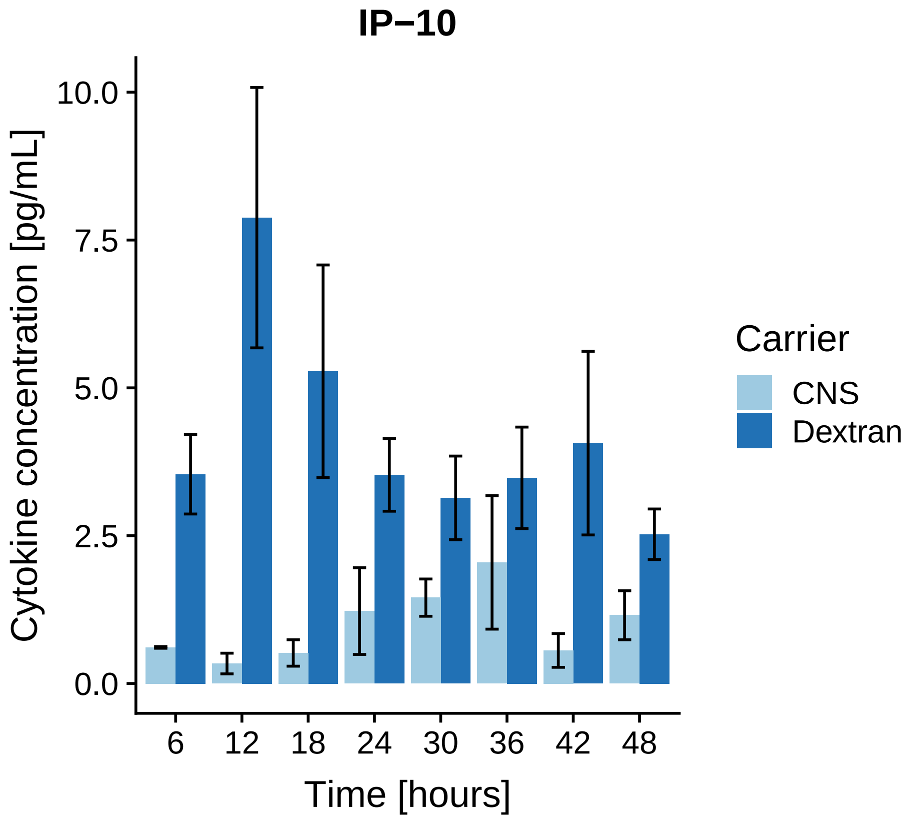
IP−10
Cytokine concentration [pg/mL]
0.0
2.5
5.0
7.5
10.0
6
12
18
24
30
36
42
48
Time [hours]
Carrier
CNS
Dextran

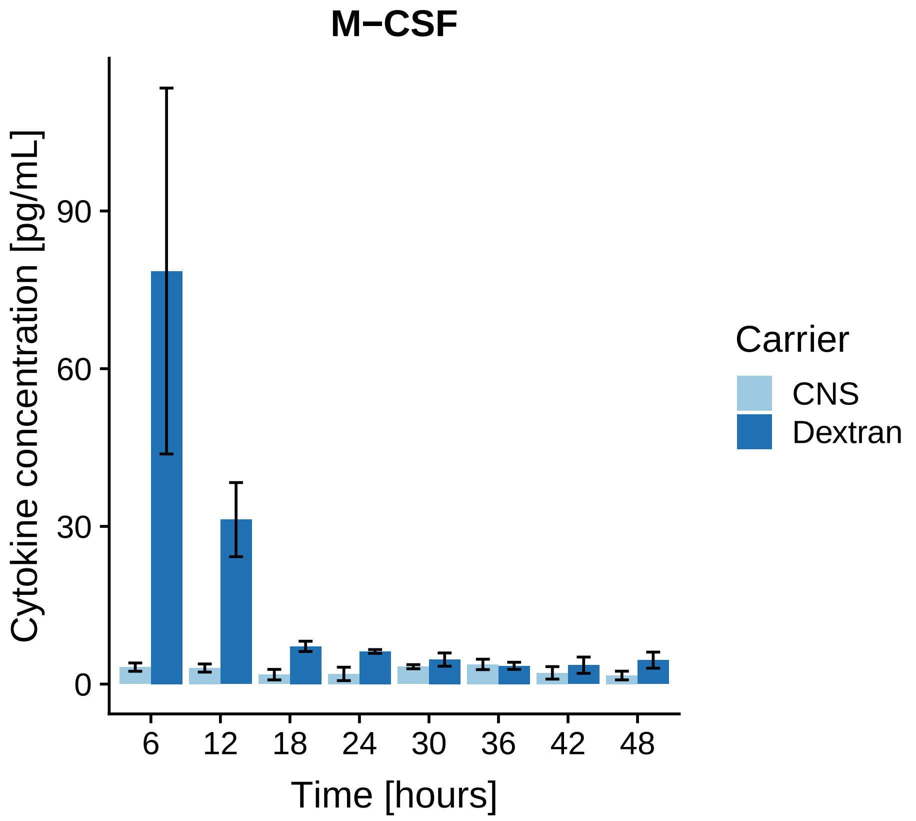
M−CSF
Cytokine concentration [pg/mL]
0
30
60
90
6
12
18
24
30
36
42
48
Time [hours]
Carrier
CNS
Dextran

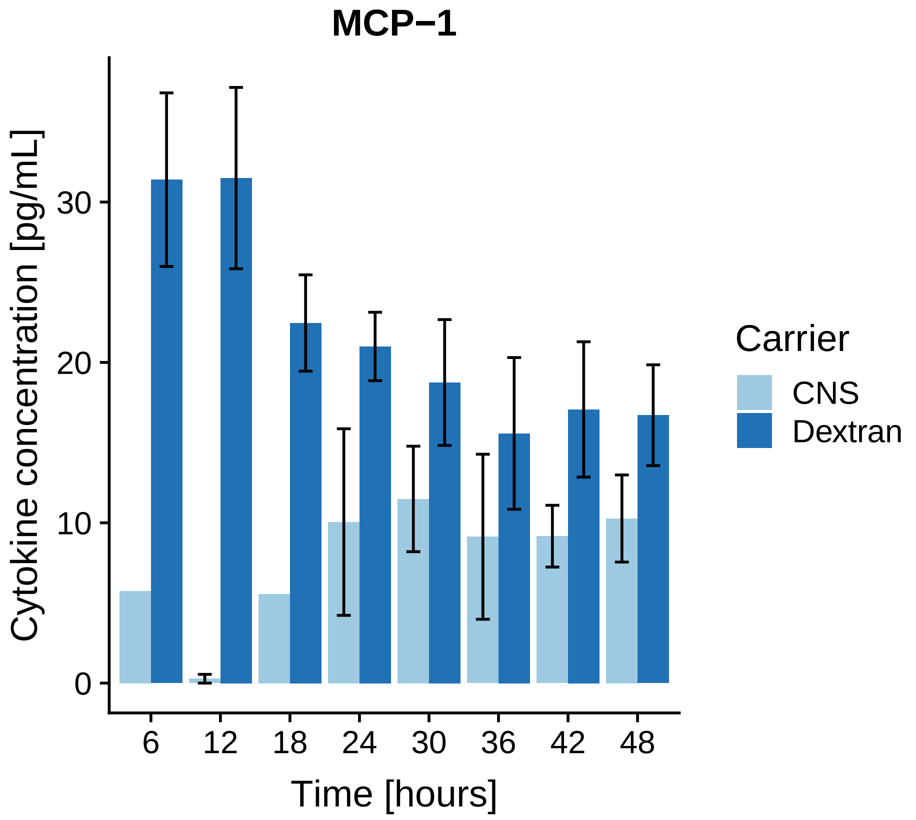
MCP−1
Cytokine concentration [pg/mL]
0
10
20
30
6
12
18
24
30
36
42
48
Time [hours]
Carrier
CNS
Dextran

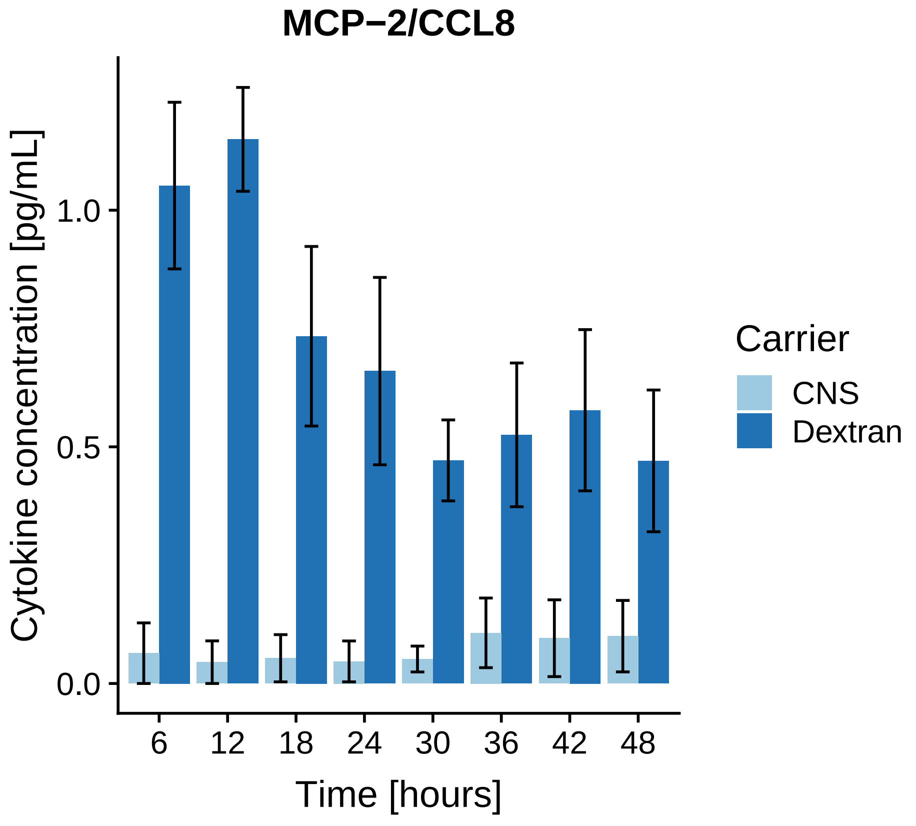
MCP−2/CCL8
Cytokine concentration [pg/mL]
0.0
0.5
1.0
6
12
18
24
30
36
42
48
Time [hours]
Carrier
CNS
Dextran

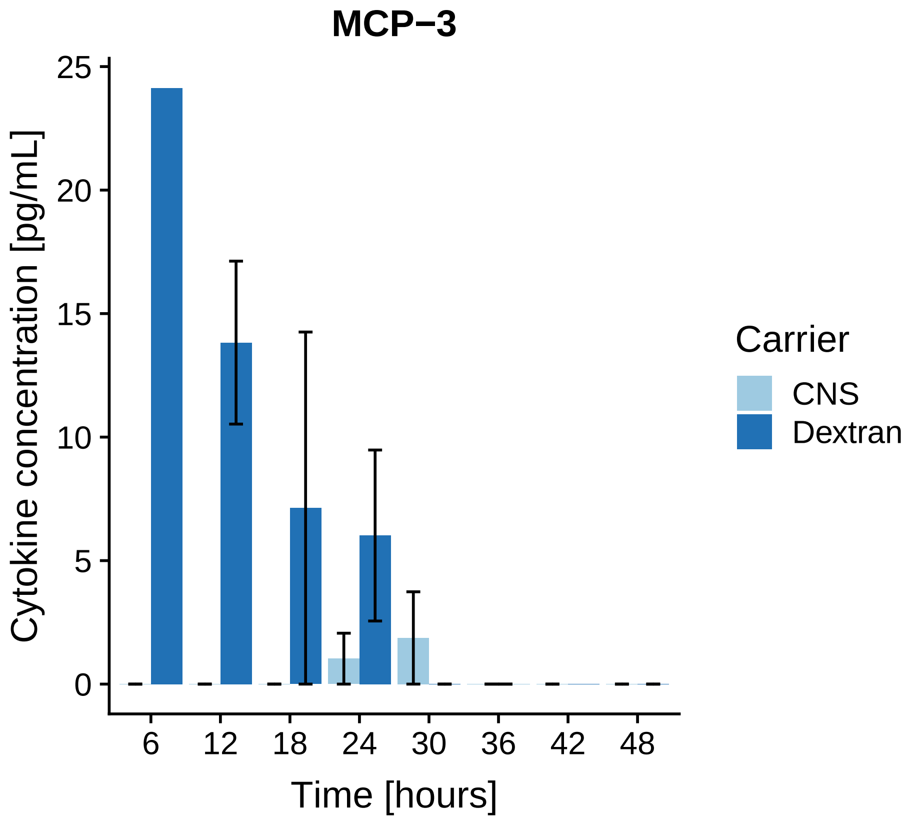
MCP−3
Cytokine concentration [pg/mL]
0
5
10
15
20
25
6
12
18
24
30
36
42
48
Time [hours]
Carrier
CNS
Dextran

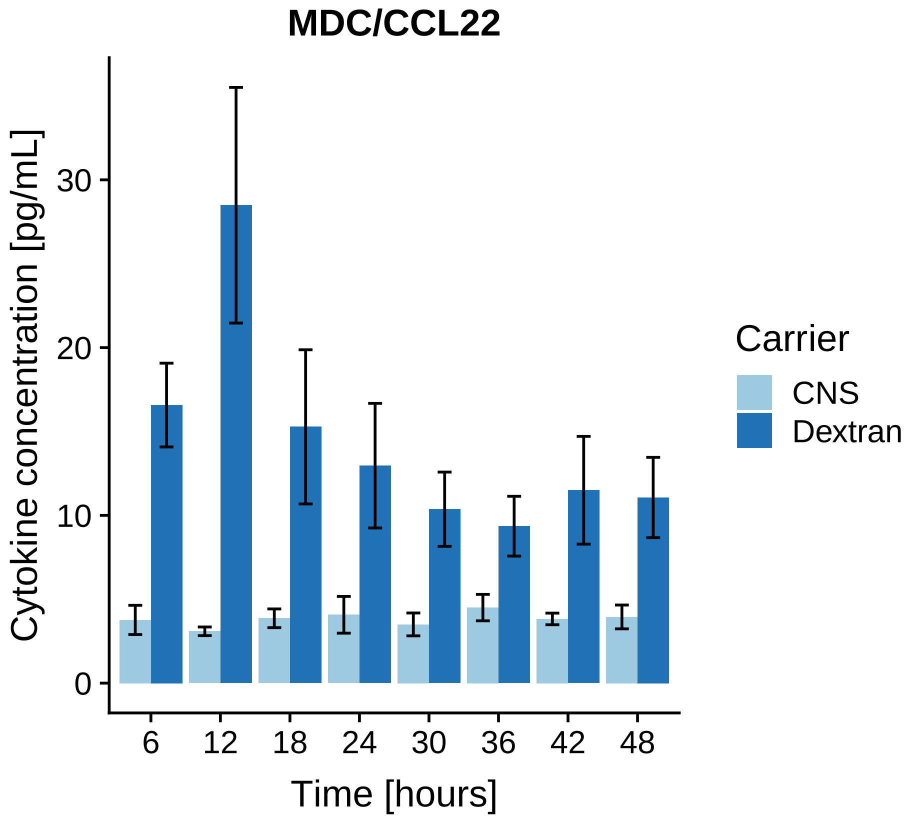
MDC/CCL22
Cytokine concentration [pg/mL]
0
10
20
30
6
12
18
24
30
36
42
48
Time [hours]
Carrier
CNS
Dextran

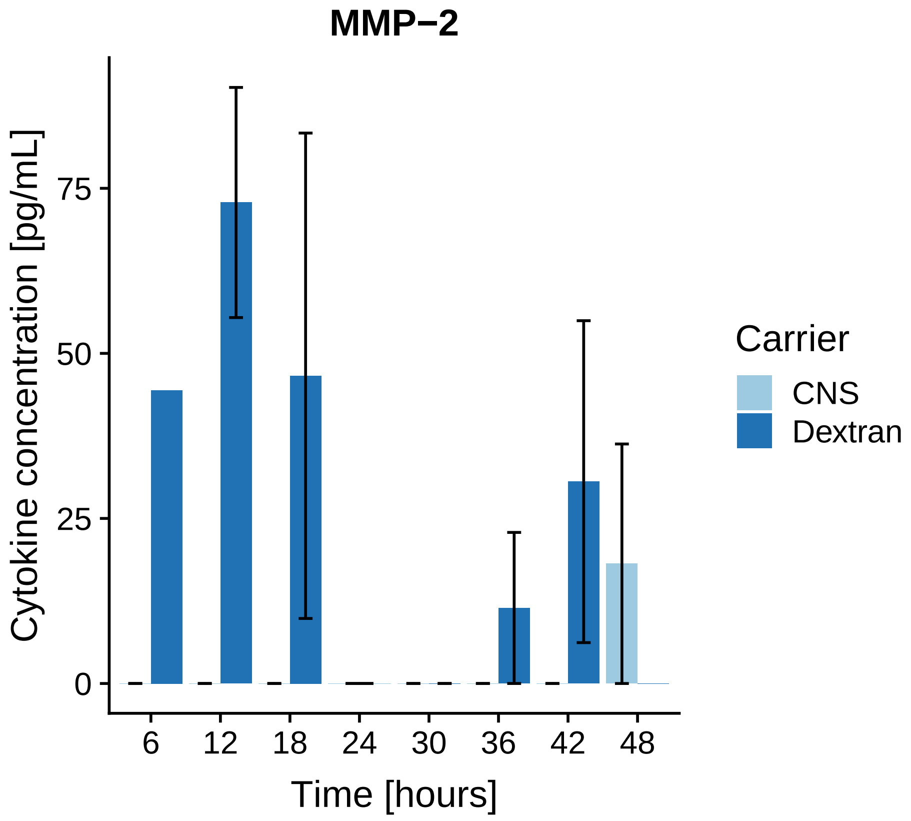
MMP−2
Cytokine concentration [pg/mL]
0
25
50
75
6
12
18
24
30
36
42
48
Time [hours]
Carrier
CNS
Dextran

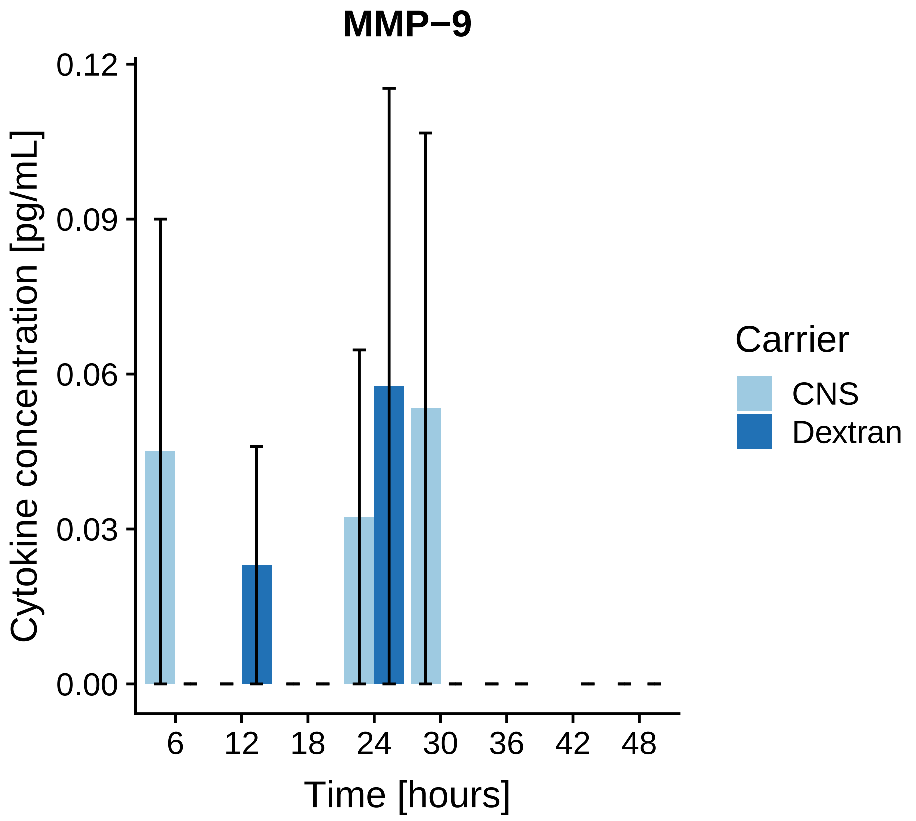
MMP−9
Cytokine concentration [pg/mL]
0.00
0.03
0.06
0.09
0.12
6
12
18
24
30
36
42
48
Time [hours]
Carrier
CNS
Dextran

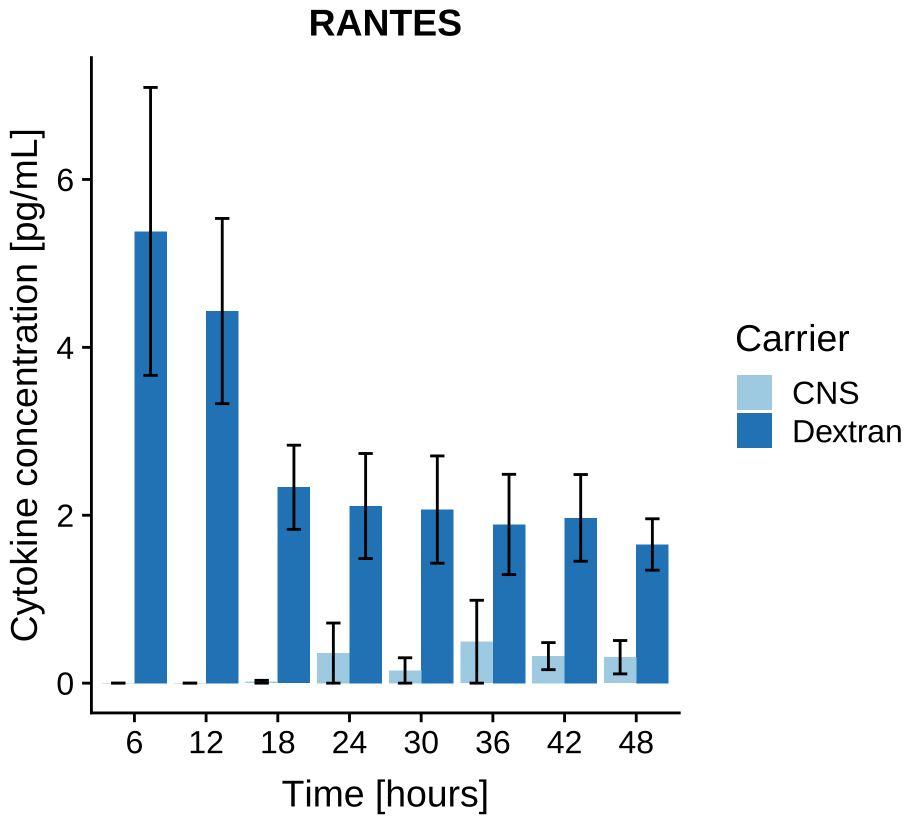
RANTES
Cytokine concentration [pg/mL]
0
2
4
6
6
12
18
24
30
36
42
48
Time [hours]
Carrier
CNS
Dextran

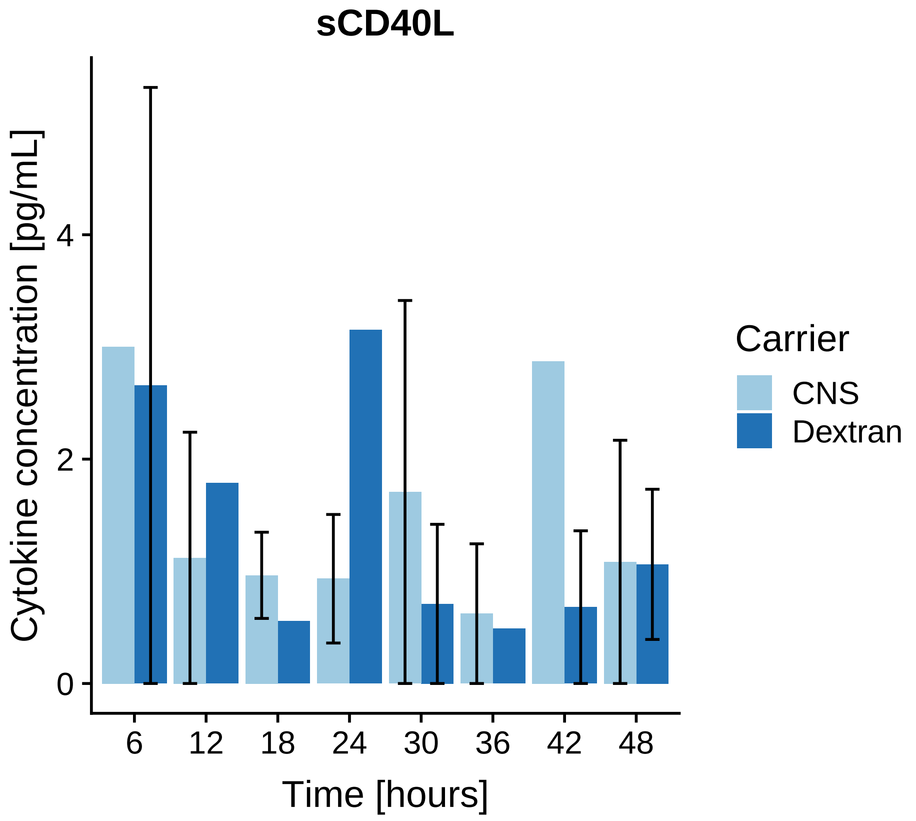
sCD40L
Cytokine concentration [pg/mL]
0
2
4
6
12
18
24
30
36
42
48
Time [hours]
Carrier
CNS
Dextran

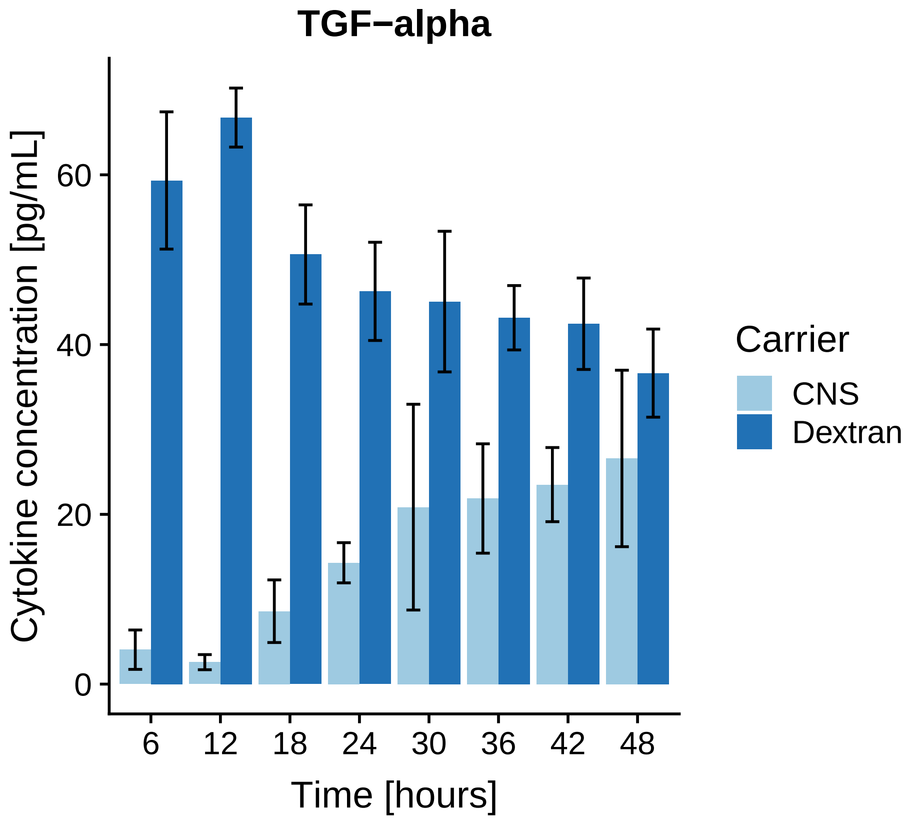
TGF−alpha
Cytokine concentration [pg/mL]
0
20
40
60
6
12
18
24
30
36
42
48
Time [hours]
Carrier
CNS
Dextran

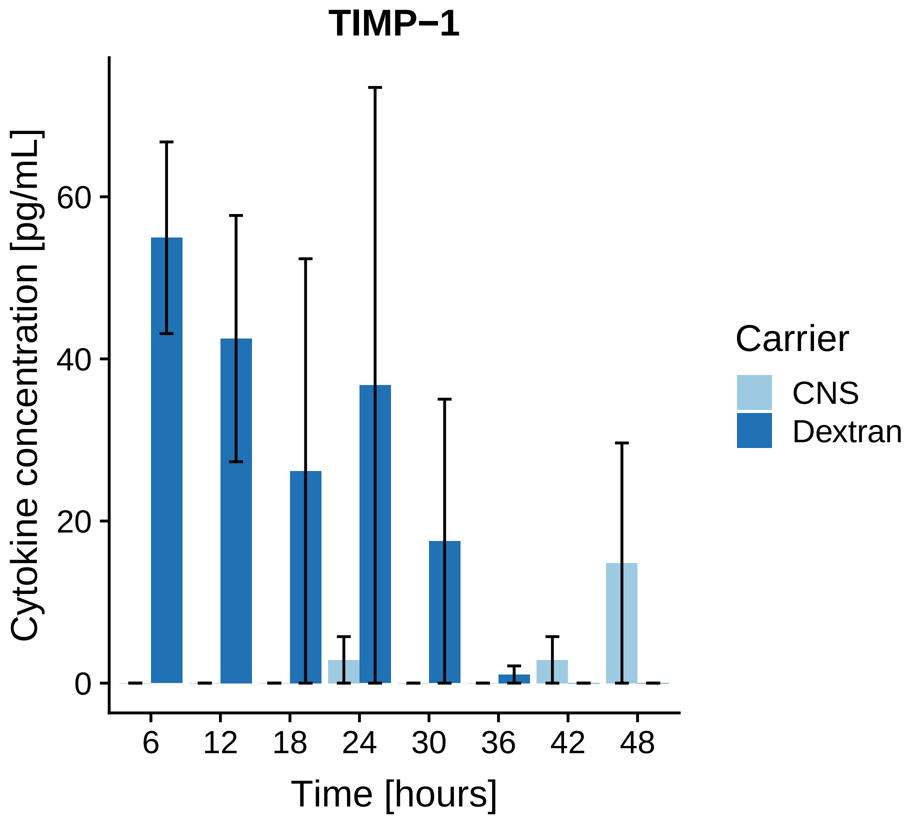
TIMP−1
Cytokine concentration [pg/mL]
0
20
40
60
6
12
18
24
30
36
42
48
Time [hours]
Carrier
CNS
Dextran

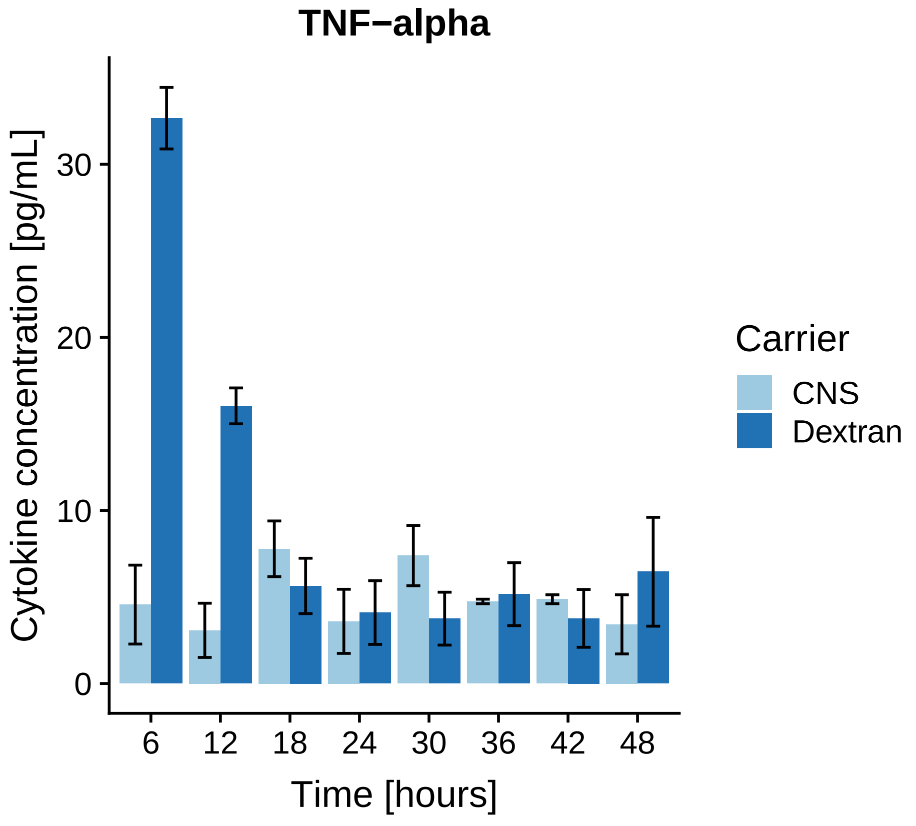
TNF−alpha
Cytokine concentration [pg/mL]
0
10
20
30
6
12
18
24
30
36
42
48
Time [hours]
Carrier
CNS
Dextran

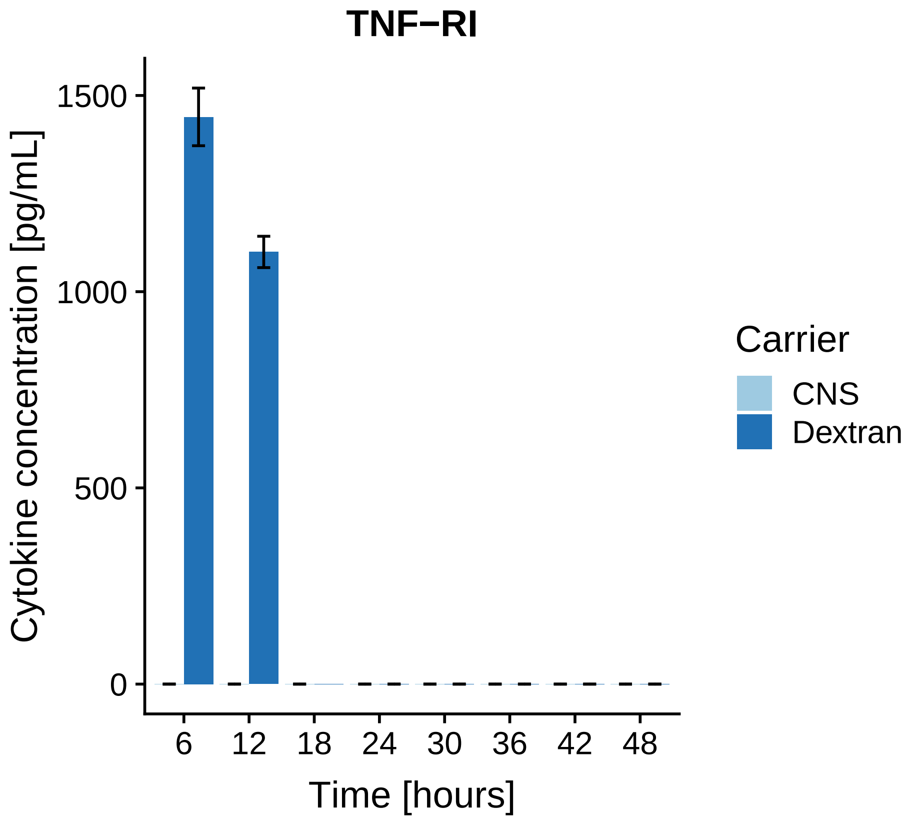
TNF−RI
Cytokine concentration [pg/mL]
1500
1000
500
0
6
12
18
24
30
36
42
48
Time [hours]
Carrier
CNS
Dextran

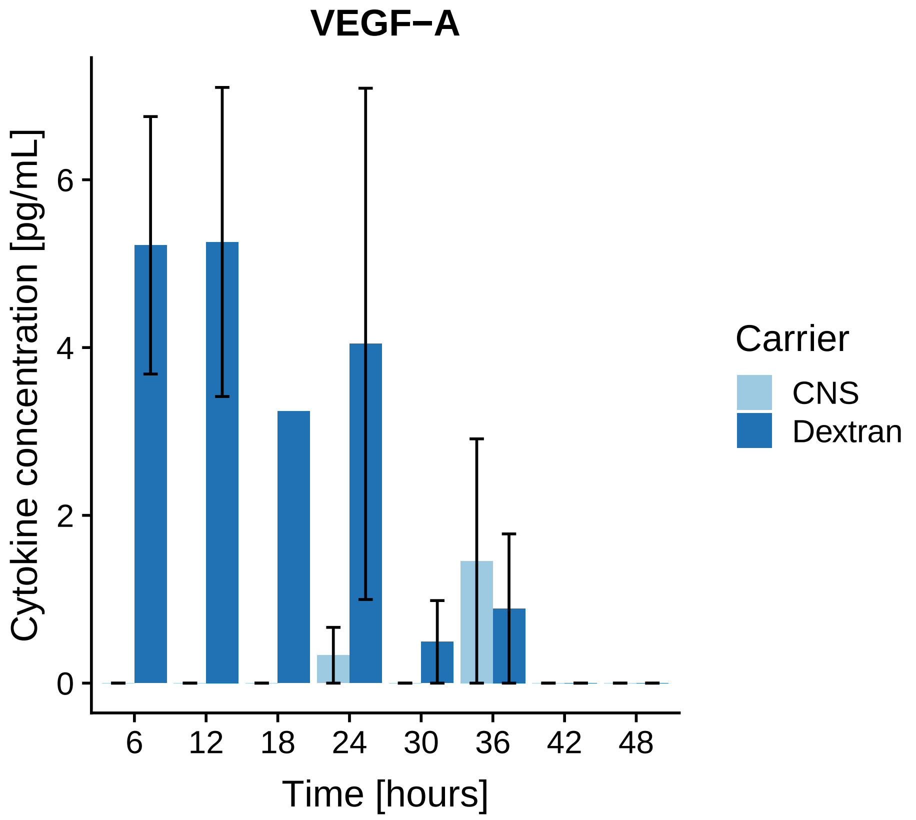
VEGF−A
Cytokine concentration [pg/mL]
6
4
2
0
6
12
18
24
30
36
42
48
Time [hours]
Carrier
CNS
Dextran

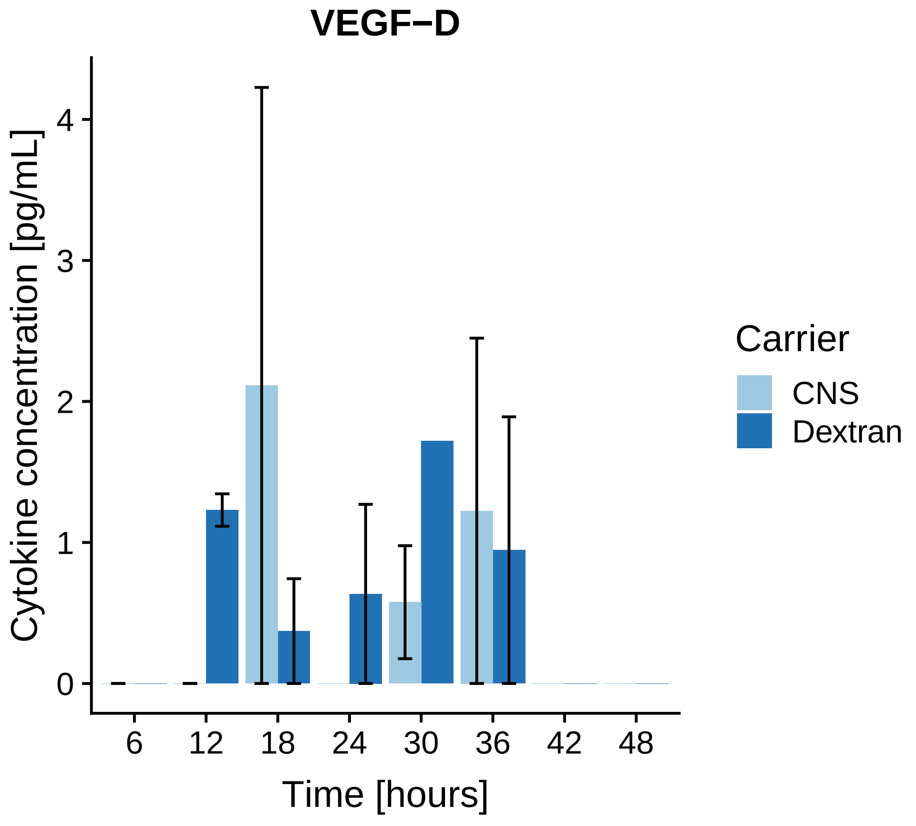
VEGF−D
Cytokine concentration [pg/mL]
4
3
2
1
0
6
12
18
24
30
36
42
48
Time [hours]
Carrier
CNS
Dextran
